# CLN7 is an organellar chloride channel regulating lysosomal function

**DOI:** 10.1126/sciadv.abj9608

**Published:** 2021-12-15

**Authors:** Yayu Wang, Wenping Zeng, Bingqian Lin, Yichuan Yao, Canjun Li, Wenqi Hu, Haotian Wu, Jiamin Huang, Mei Zhang, Tian Xue, Dejian Ren, Lili Qu, Chunlei Cang

**Affiliations:** 1 Hefei National Laboratory for Physical Sciences at Microscale, the CAS Key Laboratory of Innate Immunity and Chronic Disease, Neurodegenerative Disorder Research Center, School of Basic Medical Sciences, Division of Life Sciences and Medicine, University of Science and Technology of China, Hefei, Anhui 230026, China.; 2 CAS Key Laboratory of Brain Function and Disease, School of Life Sciences, Division of Life Sciences and Medicine, University of Science and Technology of China, Hefei, Anhui 230026, China.; 3 Department of Biology, University of Pennsylvania, Philadelphia, PA 19104, USA.

## Abstract

Neuronal ceroid lipofuscinoses (NCLs) are a group of autosomal recessive lysosomal storage diseases. One variant form of late-infantile NCL (vLINCL) is caused by mutations of a lysosomal membrane protein CLN7, the function of which has remained unknown. Here, we identified CLN7 as a novel endolysosomal chloride channel. Overexpression of CLN7 increases endolysosomal chloride currents and enlarges endolysosomes through a Ca^2+^/calmodulin-dependent way. Human CLN7 and its yeast homolog exhibit characteristics of chloride channels and are sensitive to chloride channel blockers. Moreover, CLN7 regulates lysosomal chloride conductance, luminal pH, and lysosomal membrane potential and promotes the release of lysosomal Ca^2+^ through transient receptor potential mucolipin 1 (TRPML1). Knocking out CLN7 causes pathological features that are similar to those of patients with vLINCL, including retinal degeneration and autofluorescent lipofuscin. The pathogenic mutations in CLN7 lead to a decrease in chloride permeability, suggesting that reconstitution of lysosomal Cl^−^ homeostasis may be an effective strategy for the treatment of vLINCL.

## Teaser

The disease-associated protein CLN7 forms an organellar chloride channel and regulates lysosomal calcium signaling and function.

## INTRODUCTION

Lysosomes are acidic organelles that play a vital role in eliminating worn-out proteins, damaged organelles, and other unnecessary materials in eukaryotic cells (*1*). Dysfunction of lysosomes often leads to lysosomal storage diseases (LSDs), which are characterized by abnormal accumulation of unmetabolized substrates within lysosomes (*2*). LSDs can be classified into many different subtypes based on accumulating substrate and pathological characteristics. Neuronal ceroid lipofuscinoses (NCLs) are a group of neurodegenerative LSDs that are associated with progressive symptoms including dementia, visual loss, cerebral atrophy, epilepsy, and early death (*3*). Accumulations of autofluorescent ceroid lipopigments and adenosine triphosphate (ATP) synthase subunit C are common cellular features of NCLs. Furthermore, NCLs are inherited diseases that are mostly autosomal recessive, with the exception of autosomal-dominant ceroid lipofuscinosis neuronal 4 (CLN4) (*4*). Each of the NCL subtypes is caused by mutations in one of the CLN genes. Although NCLs share common pathological and cellular characteristics, the functions of the CLN genes are diverse. For example, *CLN1*, *CLN2*, *CLN10*, and *CLN13* encode lysosomal enzymes (*5*–*9*); *CLN4* encodes a synaptic molecular machinery element protein, cysteine string protein α (*4*), while *CLN12* encodes a transporter, adenosine triphosphatase (ATPase) cation-transporting 13A2 (*10*). In addition to these well-defined *CLNs*, there are still many CLN genes with unknown functions, including *CLN7*, which we investigated in the present study.

Mutations in the *CLN7* gene cause variant late-infantile NCL (vLINCL), with an age of onset between 2 and 7 years old. The human *CLN7* gene encodes an evolutionarily conserved protein containing 518 amino acids (*11*, *12*). On the basis of homology analyses, the CLN7 protein is classified into the major facilitator superfamily (MFS) and is also referred to as MFS domain–containing 8 (MFSD8) (*11*). MFS proteins transport many substrates—such as amino acids, nucleotides, sugars, and various anions and cations (*13*)—but the potential substrate for CLN7 remains unclear. There is currently no effective treatment for vLINCL, and elucidating the function of CLN7 may facilitate the development of promising therapeutic targets.

The function of lysosomes is largely dependent on its specific ionic environment. For example, the low luminal pH caused by the accumulation of H^+^ is essential for the activity of most lysosomal enzymes. In addition, lysosomes are also important intracellular stores for Ca^2+^, Fe^2+^, and Zn^2+^. Cl^−^ is the most abundant anion in lysosomal lumen, but its role in lysosomal function and the molecular mechanism of its transport across the lysosome membrane have not been fully understood. Here, we identified CLN7 as a novel endolysosomal chloride channel and revealed its regulatory effect on lysosomal function. The pathogenic mutations in CLN7 caused a decrease in chloride permeability, suggesting that reconstitution of lysosomal Cl^−^ homeostasis may be an effective therapeutic strategy for vLINCL.

## RESULTS

### CLN7 is located in lysosomes and endosomes

**Fig. 1.**
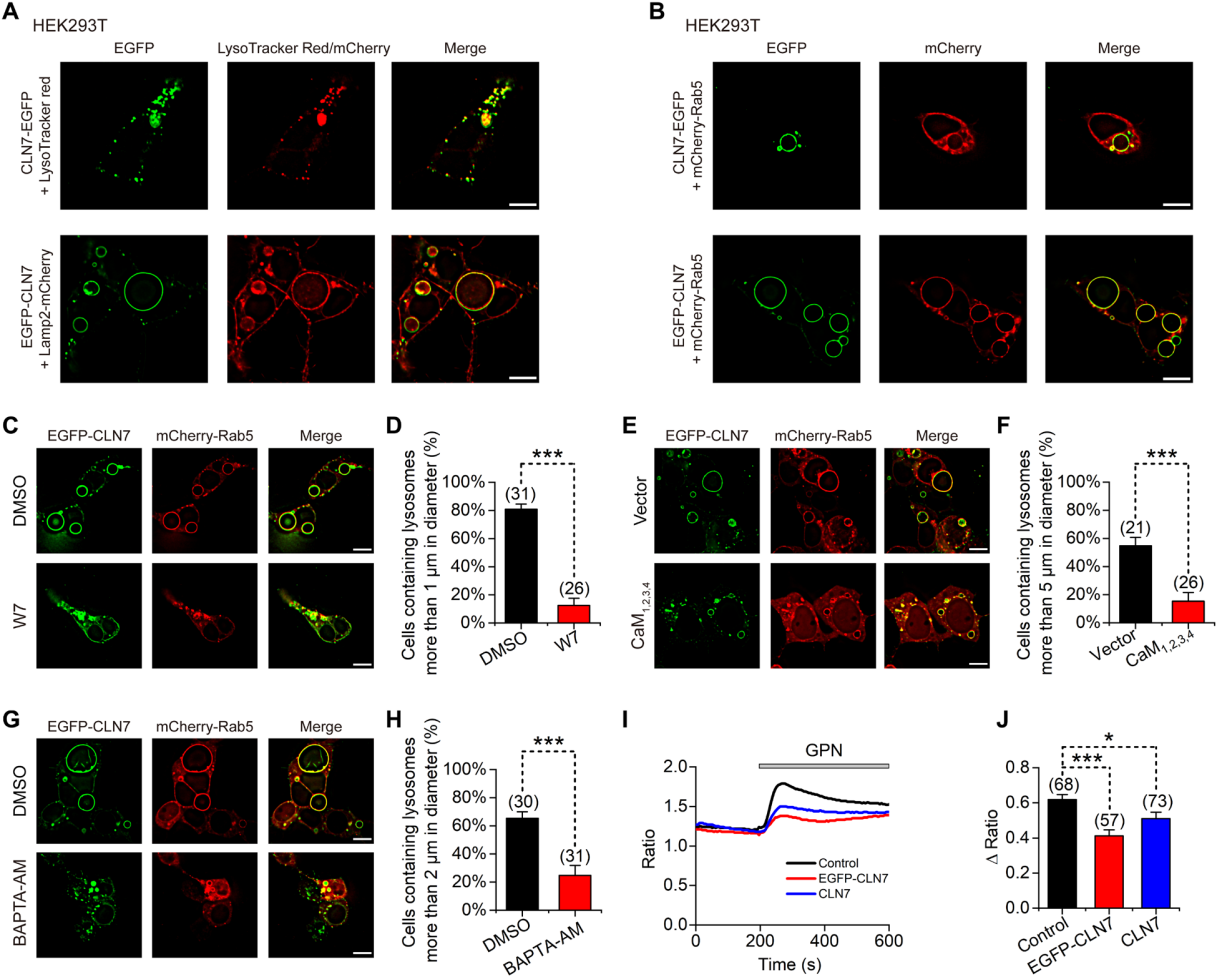
CLN7 localizes to lysosomes and endosomes and enlarges these organelles through CaM and Ca^2+^. (**A**) Colocalization of C-terminally EGFP-tagged CLN7 with LysoTracker Red (top) or N-terminally EGFP-tagged CLN7 with Lamp2-mCherry (bottom) in HEK293T cells. Scale bars, 10 μm. (**B**) Colocalization of C-terminally (top) or N-terminally (bottom) EGFP-tagged CLN7 with mCherry-Rab5 in HEK293T cells. Scale bars, 10 μm. (**C**) Fluorescence images of HEK293T cells cotransfected with EGFP-CLN7 and mCherry-Rab5. Dimethyl sulfoxide (DMSO) or 40 μM CaM inhibitor W7 was added to cells before transfection. Scale bars, 10 μm. (**D**) Percentage of cells in (C) that contain lysosomes more than 1 μm in diameter. (**E**) Fluorescence images of HEK293T cells expressing EGFP-CLN7 and mCherry-Rab5 and cotransfected with empty vector or CaM_1,2,3,4_ mutant. Scale bars, 10 μm. (**F**) Percentage of cells in (E) that contain lysosomes more than 5 μm in diameter. (**G**) Fluorescence images of HEK293T cells cotransfected with EGFP-CLN7 and mCherry-Rab5. DMSO or 50 μM BAPTA-AM was added to cells before transfection. Scale bars, 10 μm. (**H**) Percentage of cells in (G) that contain lysosomes more than 2 μm in diameter. (**I**) Representative Fura-2 fluorescence ratio (340/380) showing the effect of GPN in HEK293T cells transfected with empty vector (control), EGFP-CLN7, or nontagged CLN7. (**J**) GPN-induced changes in fluorescence ratio (340/380) of Fura-2 in cells with different transfection. Data are presented as the means ± SEM.

Before exploring the function of CLN7, we first investigated its subcellular localization. Human CLN7 (hCLN7) was tagged with enhanced green fluorescent protein (EGFP) at its N terminus or C terminus. Lysosomes were labeled with LysoTracker Red or mCherry-tagged lysosomal-associated membrane protein 2 (Lamp2), while endosomes were tracked with mCherry-tagged Rab5. Using confocal microscopy, we found that either N-terminally (EGFP-CLN7) or C-terminally (CLN7-EGFP) EGFP-tagged CLN7 colocalized with LysoTracker Red and mCherry-tagged Rab5 in transiently transfected human embryonic kidney (HEK) 293T cells (Fig. 1, A and B) and SH-SY5Y cells (fig. S1, A and B). These results suggest that hCLN7 predominantly localized to endosomes and lysosomes, which is consistent with previous studies of native proteins (*11*, *14*). Because the acidic environments of endosomal and lysosomal lumina quench EGFP fluorescence, the bright signals that we detected suggest that both the N and C termini of CLN7 were localized in the cytosol. We observed notably enlarged endolysosomes in most EGFP-CLN7–overexpressing cells. Nontagged CLN7 and CLN7-EGFP could also enlarge endolysosomes, but the effect is obviously weaker than that of EGFP-CLN7. Although slightly or moderately enlarged endolysosomes were found in most cells, notably enlarged endolysosomes were only present in a small portion of cells (fig. S2, A and B). These results suggest that the N-terminal EGFP tag may have enhanced the function of CLN7. Expressing the endosomal marker Rab5 or the lysosomal marker Lamp2 alone did not notably swell these organelles, indicating that the enlargement was caused by CLN7 (fig. S2C).

### CLN7 overexpression enlarges endolysosomes in a Ca^2+^/calmodulin-dependent way

The size of lysosomes is regulated by many factors, such as lysosomal storage, blockage of lysosomal exocytosis, and mutual fusion (*15*). The enlargement of endolysosomes caused by CLN7 overexpression was accompanied by a significant decrease in the number of endolysosomes (fig. S2, D and E), indicating that this enlargement is caused by excessive fusion of these organelles. Intracellular Ca^2+^ and calmodulin (CaM) play a vital role in organelle membrane fusion (*16*). We found that CaM inhibitor W7 largely inhibited the enlargement of endolysosomes in CLN7-overexpressing cells (Fig. 1, C and D). The enlargement was also inhibited by coexpression of a CaM mutant, CaM_1,2,3,4_ (Fig. 1, E and F) in which all four EF hands have been mutated to diminish its Ca^2+^ affinity (*17*). In addition, chelating intracellular Ca^2+^ with 1,2-Bis(2-aminophenoxy)ethane-N,N,N‘,N‘-tetraacetic acid tetrakis(acetoxymethyl ester) (BAPTA-AM) also achieved a similar effect (Fig. 1, G and H). Furthermore, we used glycyl-l-phenylalanine 2-naphthylamide (GPN) and thapsigargin (TG) to induce calcium release from lysosomes and endoplasmic reticulum, respectively, and found that the GPN-induced calcium signals were significantly reduced in cells overexpressing CLN7 (Fig. 1, I and J), while the TG-induced calcium signals remained unaffected (fig. S3, A and B). These results suggest that CLN7 promotes the release of Ca^2+^ from lysosomes and activates CaM, thereby promoting lysosomal fusion.

### CLN7 mediates endolysosomal Cl^−^ currents

**Fig. 2.**
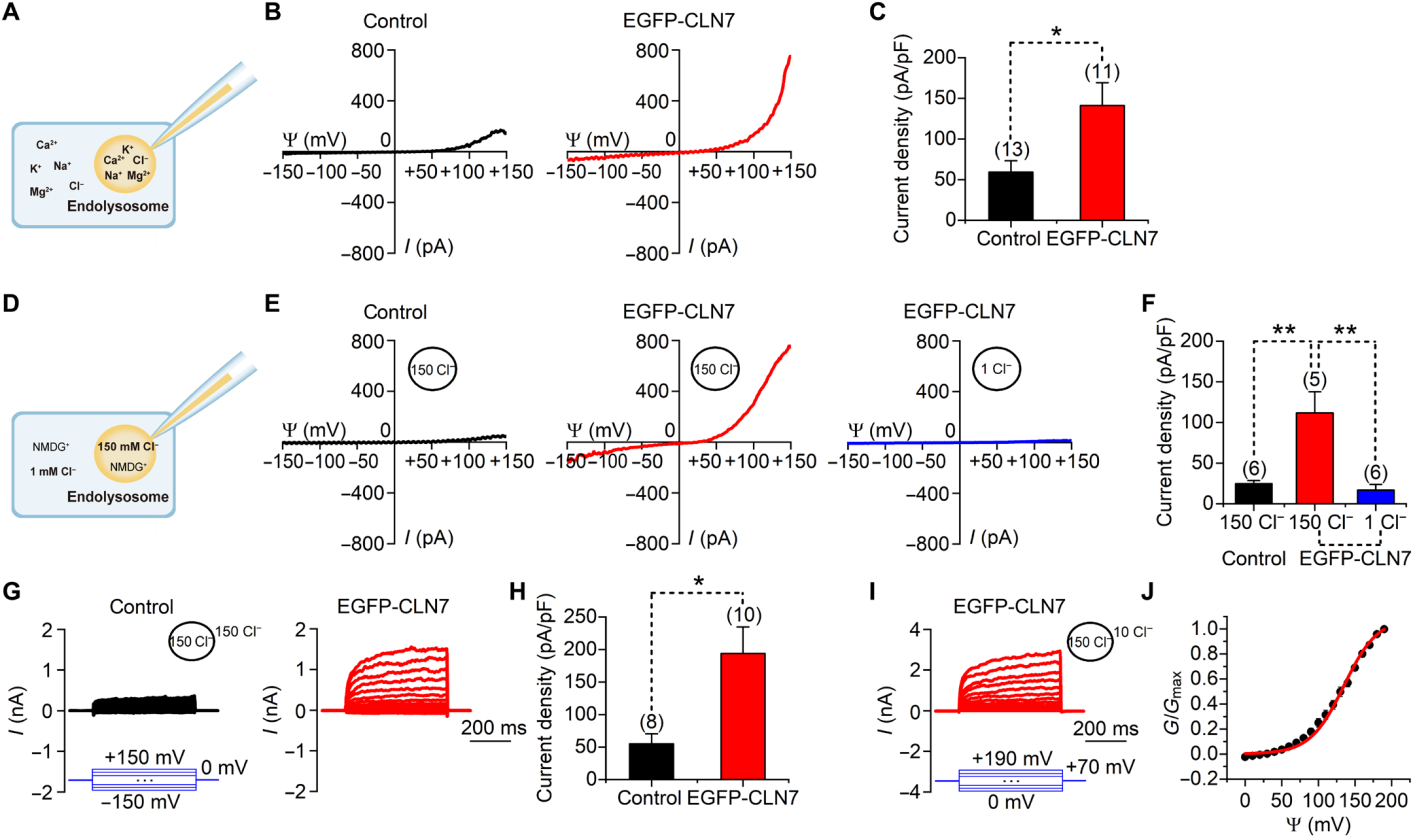
CLN7 mediates endolysosomal Cl^−^ currents. (**A** to **F**) Whole-endolysosomal currents recorded using a ramp protocol (−150 to +150 mV in 1 s, Vh = 0 mV) from nontransfected (control) or EGFP-CLN7-transfected HEK293T cells under a physiological ionic condition (A to C) or Cl^−^-based conditions with Cl^−^ as the major inorganic ion (D to F). (A and D) Illustration of the ionic conditions used in the whole-endolysosome recordings. (B and E) Representative current traces. (C and F) Current densities measured at +150 mV under conditions used in (A) and (D), respectively. (**G**) Representative whole-endolysosomal currents recorded from nontransfected (control) or EGFP-CLN7-transfected HEK293T cells at the same concentrations of Cl^−^ inside and outside of the endolysosomes (150 mM). The currents were induced using 500-ms voltage steps (−150 to +150 mV, 10 mV step; Vh = 0 mV). (**H**) Current densities measured at +150 mV under conditions used in (G). (**I**) Representative whole-endolysosomal currents recorded from EGFP-CLN7-transfected HEK293T cells under an asymmetrical Cl^−^ condition ([Cl^−^]_bath_ = 10 mM; [Cl^−^]_pipette_ = 150 mM). The currents were induced using 500-ms depolarizing steps (0 to +190 mV, 10 mV step; Vh = −70 mV). (**J**) Voltage-dependent activation curve for CLN7. Data are presented as the means ± SEM.

As a putative transporter protein on the lysosome membrane, CLN7 may directly mediate the release of Ca^2+^ from lysosome. To test this assumption, we performed whole-endolysosomal patch-clamp recordings and investigated the effect of CLN7 on ion flux across the endolysosomal membrane. We observed significantly increased outward-rectifying currents when EGFP-CLN7 was overexpressed in HEK293T cells (Fig. 2, A to C). An outward organellar current indicates a movement of positive charges into the lumen or negative charges out of the lumen. The bath and pipette solutions that we used simulated physiological ionic conditions and contained all of the major inorganic ions. After replacing the main cations (K^+^, Na^+^, Ca^2+^, and Mg^2+^) with *N*-methyl-d-glucamine ions (NMDG^+^, a large organic monovalent cation impermeable to most ion channels), we unexpectedly found that the increased currents remained unchanged (Fig. 2, D to F). The only anion in the pipette solution was Cl^−^. Furthermore, reducing the concentration of Cl^−^ in the pipette solution from 150 to 1 mM eliminated the outward current, indicating that CLN7 mediated the endolysosomal Cl^−^ conductance (Fig. 2, D to F). To further distinguish between organelles, we labeled lysosomes with Lamp2-mCherry and endosomes with mCherry-Rab5. By recording the labeled organelles, we found that Cl^−^ currents in both lysosomes and endosomes were significantly larger in CLN7-overexpressing cells than that in control cells (fig. S4). Using voltage-step protocols and a symmetrical Cl^−^ condition (i.e., 150 mM in both the bath and pipette solutions), we found that CLN7 did not conduct in negative voltages (cytosol more negative than lumen) and was slowly activated upon depolarizing the membrane potential above 0 mV (Fig. 2, G and H). The voltage dependence of CLN7 was weak in positive voltages, as can be seen from the large slop factor (31.6 ± 0.7 mV) in its activation curve (Fig. 2, I and J).

As mentioned above, fluorescent labeling may affect protein function. Hence, we next recorded the currents of CLN7-EGFP and nontagged CLN7 proteins. Similar outward-rectifying endolysosomal Cl^−^ currents were detected with these differentially tagged proteins (fig. S5). However, the current amplitude of nontagged CLN7 was smaller than that of EGFP-CLN7 but was greater than that of CLN7-EGFP. These results suggest that EGFP at the C terminus reduces the ability of CLN7 to conduct Cl^−^, while N-terminal EGFP enhances CLN7-mediated Cl^−^ conductance.

### CLN7 exhibits properties of chloride channels

**Fig. 3.**
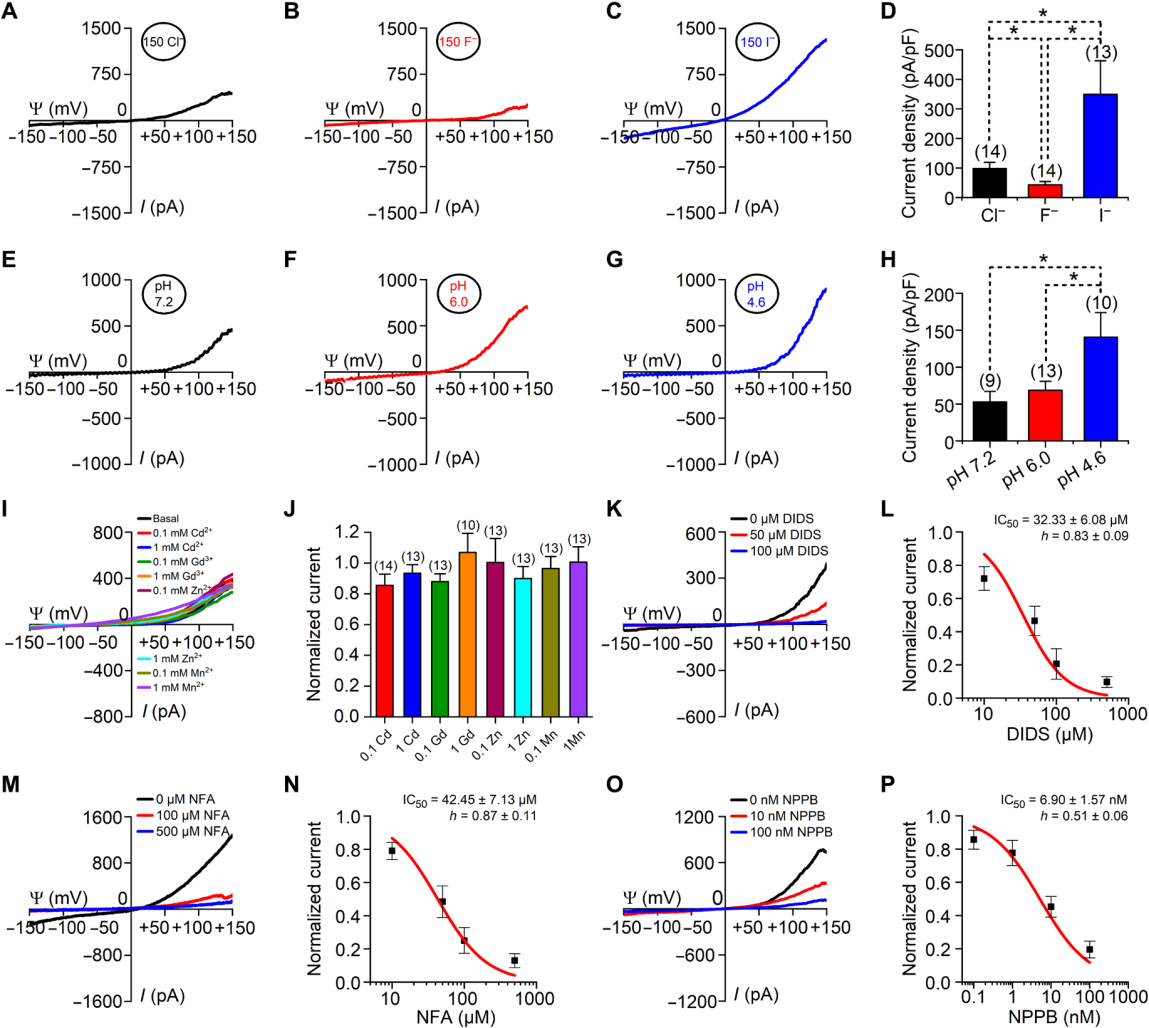
hCLN7 exhibits properties of chloride channels. Whole-endolysosomal currents were recorded using a ramp protocol (−150 to +150 mV in 1 s, Vh = 0 mV) from EGFP-hCLN7–transfected HEK293T cells. (**A** to **C**) Representative CLN7 currents recorded with pipette solutions containing 150 mM Cl^−^ (A), 150 mM F^−^ (B), or 150 mM I^−^ (C). (**D**) Current densities measured at +150 mV under conditions used in (A) to (C). (**E** to **G**) Representative CLN7 currents recorded with pipette solutions with a pH of 7.2 (E), pH of 6.0 (F), or pH of 4.6 (G). (**H**) Current densities measured at +150 mV under conditions used in (E) to (G). (**I**) Representative CLN7 currents recorded with bath solutions containing various heavy metal ions. (**J**) Current amplitudes (at +150 mV) normalized to those in the absence of heavy metal ions. (**K** to **P**) Sensitivity of CLN7 currents to DIDS (K and L), NFA (M and N), or NPPB (O and P). Representative recordings are shown in (K), (M), and (O). Dose-inhibition curves are shown in (L), (N), and (P) (*n* = 6 to 20). Data are presented as the means ± SEM.

Chloride channels are generally also permeable to other halides. To determine the permeability of CLN7, we replaced the 150 mM Cl^−^ in the pipette solution with 150 mM F^−^ or 150 mM I^−^. The currents recorded with Cl^−^ were larger than those with F^−^ but smaller than those with I^−^, indicating an approximate permeability sequence of I^−^ > Cl^−^ > F^−^ (Fig. 3, A to D). Studies have shown that some chloride channels are pH-sensitive (*18*, *19*). Considering that endosomes and lysosomes have acidic environments in their lumina, we next tested the pH sensitivity of CLN7. CLN7 currents increased upon decreases in luminal pH, indicating that CLN7 exhibited increased activity in more acidic organelles (Fig. 3, E to H). Furthermore, addition of cations (Na^+^, K^+^, Ca^2+^, and Mg^2+^) to the pipette or bath solutions had no effect on CLN7 currents, suggesting that CLN7 does not cotransport these cations (fig. S6, A and B). In addition, we found that CLN7 did not require ATP to conduct Cl^−^, as bath application of ATP failed to change CLN7 currents (fig. S6C).

Some heavy metal ions block ion channels. However, various concentrations of Cd^2+^, Gd^3+^, Zn^2+^, or Mn^2+^ did not reduce CLN7 currents (Fig. 3, I and J). 4,4‘-diisothiocyano-2,2‘-stilbenedisulfonate (DIDS), niflumic acid (NFA), and 5-Nitro-2-(3-phenylpropylamino)benzoic acid (NPPB) are three commonly used chloride channel blockers. We found that each of these blockers, when applied in bath solutions, dose-dependently blocked CLN7 currents (Fig. 3, K to P). Moreover, adding 100 μM DIDS to the culture medium largely prevented the enlargement of endolysosomes caused by overexpression of CLN7 (fig. S7). These electrophysiological and pharmacological properties suggest that CLN7 acts as a chloride channel.

So far, the three-dimensional protein structure of CLN7 has not been determined experimentally. We obtained the predicted structure of CLN7 from AlphaFold Protein Structure Database (*20*) and found four amino acid sites that are located in the potential central pore region and may be critical to the conductance of Cl^−^ (fig. S8A). To evaluate their importance to protein function, we generated five mutants of CLN7, including R465W, R465Q, R153A, Y438A, and E287A. The R465W and R465Q mutations have been found in patients with vLINCL. Using whole-endolysosomal patch-clamp recording, we found that each of these mutations significantly reduced the Cl^−^ currents compared to that of wild-type (WT) CLN7 (fig. S8, B to H). This result further supports the possibility that CLN7 is a chloride channel.

### A CLN7 mutant mediates plasma membrane Cl^−^ and I^−^ conductances

**Fig. 4.**
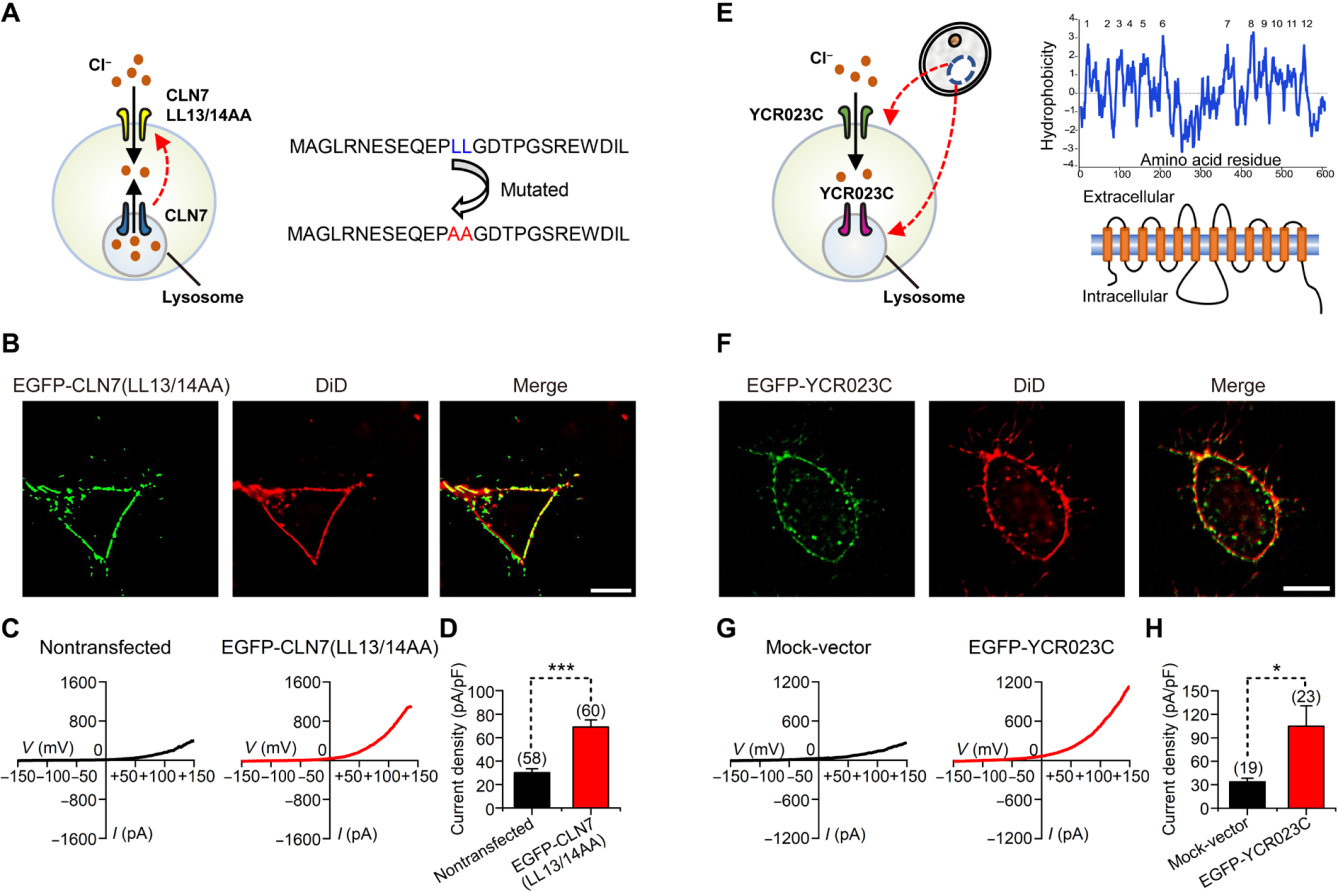
A PM-targeted CLN7 mutant and a yeast CLN7 homolog mediate whole-cell Cl^−^ currents. (**A**) Schematic diagram of PM-targeted CLN7 mutant (LL13/14AA). (**B**) Colocalization of EGFP-CLN7 (LL13/14AA) and DiD in HeLa cells. Scale bar, 10 μm. (**C**) Whole-cell currents recorded from nontransfected or EGFP-CLN7 (LL13/14AA)–transfected HeLa cells. (**D**) Current densities measured at +150 mV in cells used in (C). (**E**) Schematic diagram of the yeast CLN7 homolog, YCR023C (left), and hydrophobicity and predicted 12-transmembrane topology of YCR023C (right). (**F**) Colocalization of EGFP-tagged YCR023C and DiD in HeLa cells. Scale bar, 10 μm. (**G**) Whole-cell currents recorded from mock-transfected or EGFP-tagged YCR023C-transfected HeLa cells. (**H**) Current densities measured at +150 mV in cells used in (G). Data are presented as the means ± SEM.

Lysosomal targeting of CLN7 requires a dileucine-based sorting motif in its N terminus (*21*). Replacing the leucines with alanines (LL13/14AA) redirects CLN7 to the plasma membrane (PM), as shown by the colocalization of CLN7 and the PM-specific binding dye, 1,1‘-Dioctadecyl-3,3,3‘,3‘-tetramethylindodicarbocyanine perchlorate (DiD), in our present study (Fig. 4, A and B). To test the function of the PM-targeting CLN7 mutant, we cotransfected N-terminally mCherry-tagged CLN7-LL13/14AA with an iodide-sensitive EYFP-H148Q/I152L variant (*22*) into HEK293T cells. The expression of CLN7-LL13/14AA significantly increased the sensitivity of intracellular enhanced yellow fluorescent protein (EYFP) fluorescence to extracellular application of NaI, indicating that CLN7-LL13/14AA mediated the transportation of iodide across the PM (fig. S9, A and B). Next, we performed whole-cell recordings in HeLa cells and found that the Cl^−^ currents were significantly larger in EGFP-CLN7-LL13/14AA–overexpressing cells than that in nontransfected cells (Fig. 4, C and D). Similar to the properties of endolysosomal CLN7 currents, the whole-cell currents were also outward-rectifying and sensitive to pH, DIDS, NFA, and NPPB (fig. S9, C to E). These results indicate that CLN7 function does not depend on other molecules specific to the endolysosomal membrane.

### The yeast CLN7 homolog mediates PM Cl^−^ conductance

CLN7 is highly conserved across species (*11*). The yeast CLN7 homolog, YCR023C (from *Saccharomyces cerevisiae*), is also predicted to have 12 membrane-spanning domains (Fig. 4E). When expressed in HeLa cells, YCR023C was found in both PM and intracellular components (Fig. 4F). The expression of YCR023C also increased the whole-cell Cl^−^ currents (Fig. 4, G and H). In addition, the whole-cell currents recorded in YCR023C-expressing cells had the similar characteristics as those of hCLN7 currents, including outward rectification and sensitivities to DIDS, NFA, and NPPB (fig. S9F). Since other lysosomal ion channels or transporters are usually not expressed on the PM, and YCR023C is less than 25% identical to that of hCLN7 and therefore unlikely to serve as an auxiliary subunit for a human protein, the data from both the PM-targeting CLN7 mutant and the yeast CLN7 homolog suggest that CLN7 is more like a Cl^−^-conducting protein than as an auxiliary subunit.

### CLN7 is a primary mediator of endolysosomal Cl^−^ conductance

**Fig. 5.**
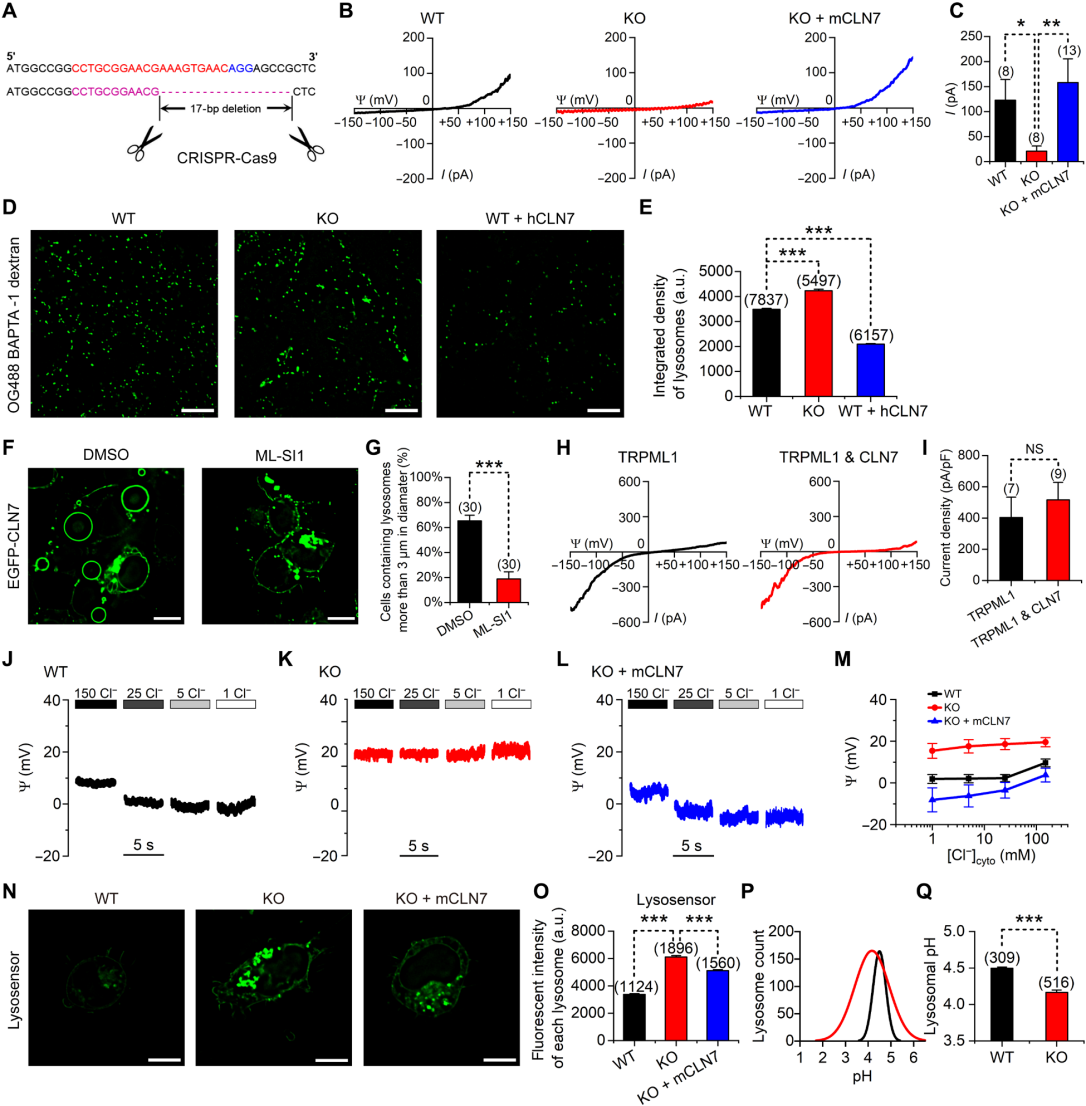
CLN7 regulates lysosomal chloride conductance, luminal calcium, membrane potential, and luminal pH. (**A**) Schematic diagram showing CLN7-KO in HEK293T cells. (**B** and **C**) Representative traces (B) and amplitudes (C, at +150 mV) of lysosomal Cl^−^ current recorded in WT, CLN7-KO, and mCLN7-transfected CLN7-KO cells. (**D**) WT, CLN7-KO, and CLN7-overexpressing HEK293T cells loaded with OG488-BAPTA-1-dextran. (**E**) Integrated fluorescent intensities of lysosomes in cells represented in (D). (**F**) EGFP-CLN7–overexpressing cells treated with DMSO or 20 μM ML-SI1 (added to cells before transfection). (**G**) Percentage of cells represented in (F) containing lysosomes larger than 3 μm. (**H** and **I**) Representative traces (H) and current densities (I, at −150 mV) of whole-endolysosomal currents recorded in HEK293T cells transfected with TRPML1 alone or cotransfected with CLN7. (**J** to **L**) Representative lysosomal membrane potentials recorded under various [Cl^−^]_bath_ using current-clamp (*I* = 0) from WT, CLN7-KO, and mCLN7-transfected-CLN7-KO cells. (**M**) Lysosomal membrane potentials measured from cells represented in (J) to (L). (**N**) LysoSensor staining in WT, CLN7-KO, and mCLN7-transfected CLN7-KO cells. (**O**) Averaged fluorescent intensities of lysosomes in cells represented in (N). (**P** and **Q**) Quantification of lysosomal pH measured using OG488–dextran in WT or CLN7-KO cells. Scale bars, 10 μm. Data are presented as the mean ± SEM. NS, not significant; a.u., arbitrary units.

To explore the function of CLN7 in lysosomal physiology, we used the CRISPR-Cas9 technique to knockout (KO) CLN7 in HEK293T cells. A single-guide RNA (sgRNA) targeting protospacer-adjacent motif (PAM)–adjacent sequences in the second exon of CLN7 was used. A 17-bp deletion was generated in the KO cells, which caused a frame-shift mutation and protein truncation (Fig. 5A). By performing whole-endolysosomal recordings, we found that loss of hCLN7 largely reduced endolysosomal Cl^−^ currents, whereas transfection of mouse CLN7 (mCLN7) rescued these currents (Fig. 5, B and C). In addition, we knocked down hCLN7 in HEK293T cells via short hairpin RNA (shRNA) and found that this manipulation reduced Cl^−^ currents (fig. S10, A to C). Therefore, we conclude that CLN7 is a primary mediator of endolysosomal Cl^−^ conductance.

### CLN7 regulates lysosomal calcium content

We have shown that CLN7 overexpression regulates calcium and CaM signaling (Fig. 1, C to J). Here, we used the CLN7-KO cells to further study CLN7’s effect on lysosomal calcium content. We loaded the lysosomal calcium indicator Oregon Green 488 (OG488) BAPTA-1 dextran to WT, CLN7-KO, and CLN7-overexpressing HEK293T cells. Compared to that in WT cells, the lysosomal calcium signal was significantly increased in CLN7-KO cells but decreased in CLN7-overexpressing cells (Fig. 5, D and E).

As CLN7 itself is a chloride channel, its regulation of lysosomal calcium must be indirect and requires the participation of other proteins. TRPML1 is the main Ca^2+^-permeable channel on the lysosomes. A TRPML1 inhibitor ML-SI1 significantly reduced lysosomal enlargement caused by CLN7 overexpression (Fig. 5, F and G), indicating that CLN7 regulates lysosomal size and the luminal calcium through TRPML1. However, in the lysosomal patch-clamp recording, we found that overexpression of CLN7 does not affect TRPML1 current (Fig. 5, H and I). TRPML1 is a voltage-sensitive channel that can be activated by a negative voltage (i.e., the voltage on the cytoplasmic side is lower than that on the lysosome luminal side). In the patch-clamp experiment, we “clamped” the membrane at desired voltage values. The expression of CLN7 probably affected neither the expression level of TRPML1 nor its voltage dependence, so we could not detect changes in TRPML1 current in our patch-clamp experiment. In live cells, CLN7 may affect the function of TRPML1 by affecting the lysosomal membrane potential.

### CLN7 regulates the lysosomal membrane potential

Membrane permeability to ions is one of the core factors that affects membrane potential. As the most abundant anion in the lysosomal lumen, Cl^−^ and its conducting protein, CLN7, are likely to have a notable effect on the lysosomal membrane potential. To test this hypothesis, we performed current-clamp recordings in endolysosomes. Compared to that in WT cells, the lysosomal membrane potential in CLN7-KO cells was elevated and less sensitive to changes in Cl^−^ concentrations (Fig. 5, J to M). Transfecting mCLN7 into CLN7-KO cells decreased the lysosomal membrane potential and restored its sensitivity to changes in Cl^−^ concentrations (Fig. 5, L and M). Combined with the results of Fig. 5 (D to I), we can speculate that CLN7 reduces the lysosomal membrane potential, which, in turn, promotes the activation of TRPML1 and further promotes the release of lysosomal calcium ions.

### CLN7 regulates lysosomal pH

Cl^−^ has long been considered as the counterion for lysosomal proton (H^+^) pumps (*23*). The progressive acidification from endosomes to lysosomes is accompanied by an increase in Cl^−^ concentration (*24*, *25*). On the basis of these previous studies, we speculated that CLN7 is involved in regulating lysosomal pH. In CLN7-KO and CLN7-knockdown HEK293T cells, decreases in luminal pH were detected using the lysosomal pH indicators, LysoSensor Green and OG488–dextran (Fig. 5, N to Q, and fig. S10, D and E). The mean pH values were 4.50 in WT cells and 4.17 in KO cells. Similarly, knockdown of CLN7 in HeLa and SH-SY5Y cells also decreased the lysosomal pH (fig. S11). This decrease in endolysosomal pH was partially rescued by transfecting mCLN7 into KO cells (Fig. 5, N and O). Collectively, these results confirmed the involvement of CLN7 in the regulation of lysosomal pH.

### Pathogenic mutations in CLN7 affect Cl^−^ currents

**Fig. 6.**
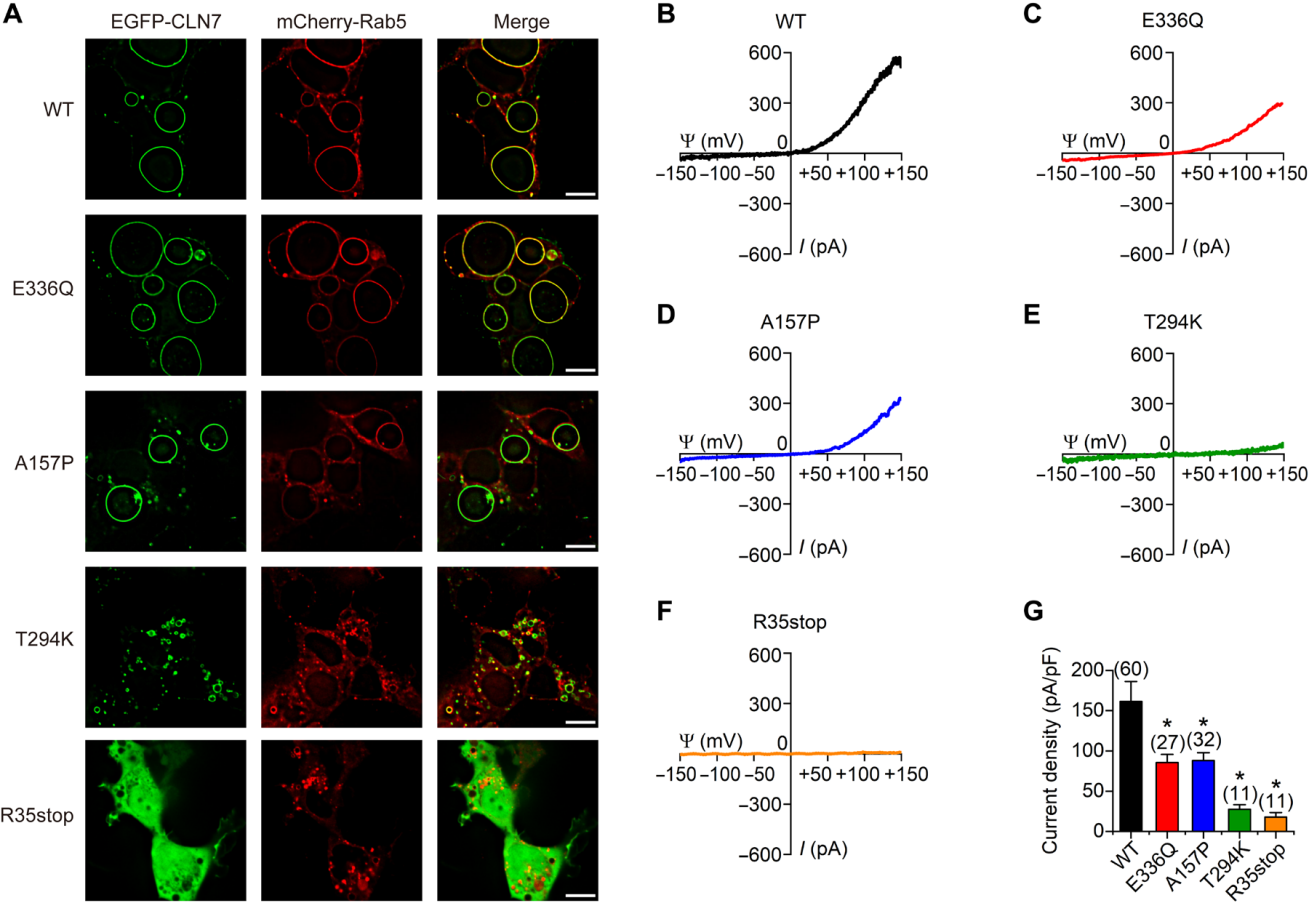
Pathogenic mutants of CLN7 exhibits impaired chloride conductance. (**A**) Colocalization of N-terminally EGFP-tagged WT and mutated hCLN7 with mCherry-tagged Rab5 in HEK293T cells. Scale bars, 10 μm. (**B** to **F**) Representative endolysosomal currents recorded in HEK293T cells transfected with WT hCLN7 (B), hCLN7-E336Q (C), hCLN7-A157P (D), hCLN7-T294K (E), or hCLN7-R35stop (F). (**G**) Statistics of current densities of (B) to (F). Data were presented as means ± SEM.

*CLN7* has been found to be the causative gene for vLINCL. E336Q, A157P, T294K, and R35stop are four of many known pathogenic mutations found in patients with vLINCL (mutations can be found in the NCL Resource at http://ucl.ac.uk/ncl/). The T294K and R35stop mutations cause more severe clinical symptoms compared to those from E336Q and A157P mutations. To test the effects of these four pathogenic mutations on endolysosomal Cl^−^ currents, HEK293T cells were cotransfected with mCherry-Rab5 and EGFP-tagged each of these mutations. As shown in Fig. 6A, E336Q, A157P, and T294K displayed considerable colocalization with endolysosomes, while R35stop was distributed diffusely throughout the cytosol. Using electrophysiological recordings of whole-endolysosomal currents, we found that each of the mutations reduced Cl^−^ currents compared to that of WT CLN7. Moreover, the T294K and R35stop mutations, which are known to result in more severe symptoms, exhibited more serious defects in chloride channel function compared to those from E336Q and A157P (Fig. 6, B to G). These findings may provide a partial mechanistic explanation for the differential degrees of clinical symptoms among these four CLN7 mutations. A lack of enlarged endolysosomes in T294K- and R35stop-transfected cells further indicates a disruption of protein function and a linkage between Cl^−^ homeostasis and organellar morphology. The E336Q and A157P mutations eliminated the pH sensitivity of the CLN7 (fig. S12, A, B, D, and E) but did not significantly alter its voltage sensitivity (fig. S12, C and F). Meanwhile, these two mutations also altered the selectivity of CLN7 to halide ions (fig. S13, compared to Fig. 3, A to D).

### CLN7-KO mice recapitulate pathological features of human NCL

**Fig. 7.**
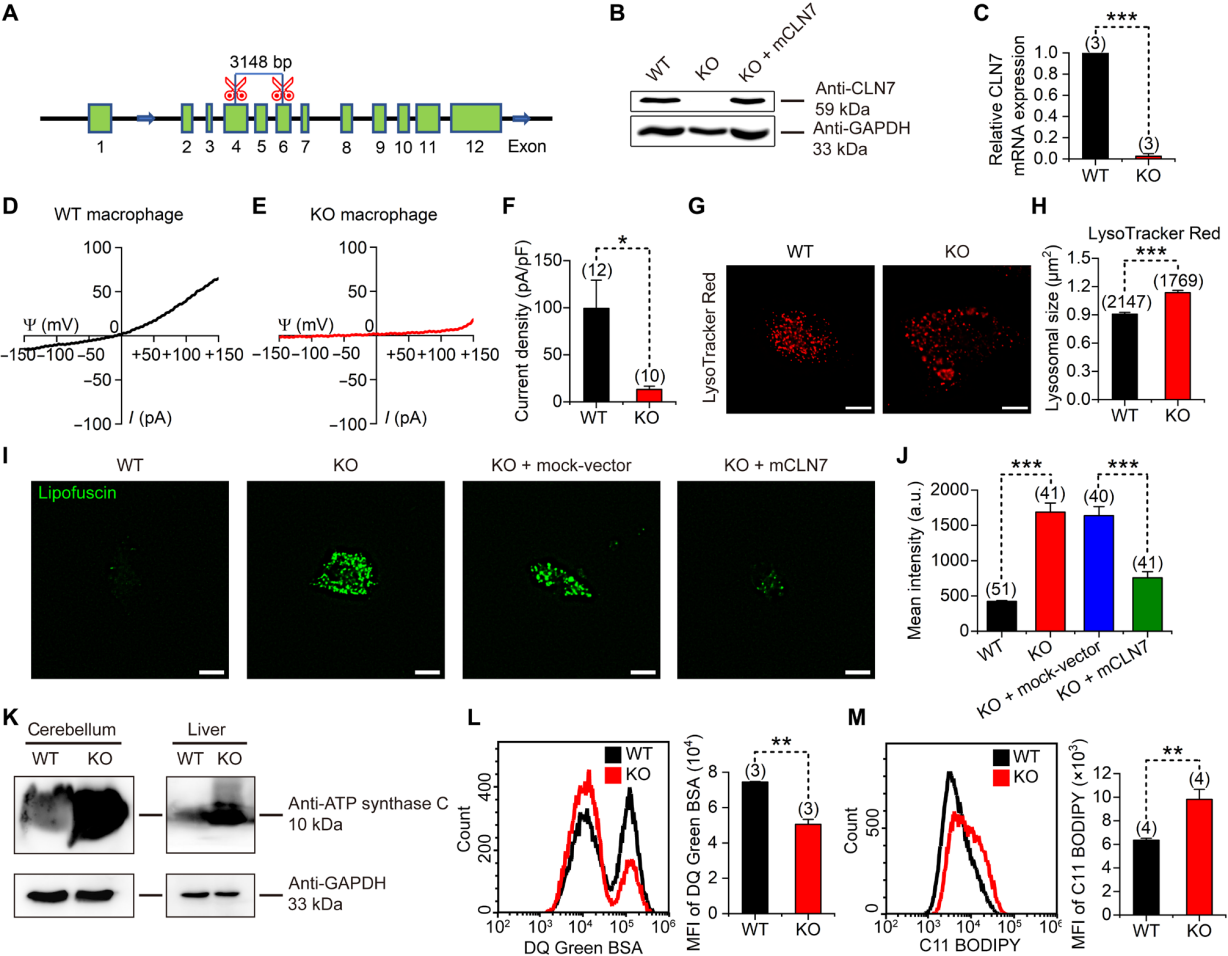
CLN7-KO mice recapitulate pathological features of human vLINCL. (**A**) Schematic diagram showing the KO strategy of *CLN7* in C57BL/6N mice. A 3148-bp segment was deleted from the *CLN7* locus. (**B**) Western blot analysis of CLN7 protein levels in MEFs from WT, CLN7-KO, and CLN7-KO MEF cells following mCLN7 transfections. (**C**) Relative CLN7 mRNA level in MEF cells derived from WT and CLN7-KO mice. (**D** and **E**) Whole-endolysosomal Cl^−^ currents recorded from peritoneal macrophages derived from WT (D) or CLN7-KO mice (E). (**F**) Current densities measured at +150 mV in cells represented in (D) and (E). (**G**) Representative fluorescence images of WT and CLN7-KO MEF cells stained with LysoTracker Red. Scale bars, 10 μm. (**H**) Lysosome size shown as the area of the lysosomes in cells represented in (G). For WT cells, *n* = 2147 lysosomes from 44 cells performed on one passage. For KO cells, *n* = 1769 lysosomes from 43 cells performed on one passage. (**I**) Autofluorescent imaging of lipofuscin in WT or CLN7-KO MEFs (P11) and CLN7-KO cells that received different transfections (mock-vector or mCLN7). Scale bars, 10 μm. (**J**) Statistics of mean fluorescent intensities of (I). (**K**) Western blot analysis of accumulated mitochondrial ATP synthase subunit C in mouse cerebellums and livers. (**L**) Flow cytometry analysis of lysosomal degradation ability in WT and CLN7-KO MEF cells loaded with DQ Green BSA. (**M**) Flow cytometry analysis of C11 BODIPY staining in WT and CLN7-KO MEF cells. Data are presented as the means ± SEM. MFI, mean fluorescence intensity.

Similar to hCLN7, mCLN7 also enlarged endolysosomes and increased endolysosomal Cl^−^ currents when expressed in HEK293T cells (fig. S14). To explore the function of CLN7 in vivo, we generated a CLN7-KO mouse line via CRISPR-Cas9 technology. Two sgRNAs targeting the fourth and sixth exons were used, resulting in deletion of 3148 bp of the genome segment of the *CLN7* gene (Fig. 7A). Successful KO of CLN7 was verified by using Western blot and real-time quantitative polymerase chain reaction (RT-qPCR) to detect protein and mRNA levels in primary cultured mouse embryonic fibroblasts (MEFs) (Fig. 7, B and C) and via recording of whole-endolysosomal Cl^−^ currents in cultured peritoneal macrophages (Fig. 7, D to F). Overexpression of CLN7 has been shown to enlarge lysosomes (Fig. 1). However, in the CLN7-KO MEF cells, we were surprised to find that the size of the lysosome was not smaller but rather larger than that of the WT cell (Fig. 7, G and H). Similar result was obtained in CLN7-KO HEK293T cells (fig. S15, A and B). This may be due to lysosomal storage caused by CLN7-KO.

Degradation of abnormal cell contents in lysosomes is a key factor to maintain cellular proteostasis. In NCL diseases, as a consequence of the impaired lysosomal function, highly cross-linked lipofuscin (the main components are lipids, metals, misfolded proteins, and sugar residues) accumulate in lysosomes, leading to autofluorescence and lipid peroxidation. In addition, mitochondrial components delivered to lysosomes through mitophagy, such as ATP subunit C, also aggregate in lysosomes. In late-passage (P11) MEFs, we found that intracellular autofluorescent levels in CLN7-KO cells were significantly higher than those in WT cells (Fig. 7, I and J). Transfecting CLN7 back into CLN7-KO MEFs significantly reduced intracellular autofluorescent levels (Fig. 7, I and J). Higher level of autofluorescence was also detected in the hippocampus and cerebellum of CLN7-KO mice (fig. S16). Moreover, 4- to 6-month-old CLN7-KO mice exhibited strong retinal degeneration (fig. S17, A to D) and poor performance in the visual water maze (fig. S17, E to G), which is consistent with the pathological features of blindness in patients with vLINCL. In addition, the cerebellar and liver tissues from 6-month-old CLN7-KO mice had increased accumulation of ATP synthase subunit C compared to that in WT mice (Fig. 7K). These pathological features are similar to those found in another CLN7-KO mouse model (*26*, *27*). In the CLN7-KO MEF cells, we also detected a decrease in the ability of lysosomes to degrade proteins (Fig. 7L). In addition, KO cells showed higher levels of lipid peroxidation (Fig. 7M), which may be an important factor leading to cell death in CLN7 disease.

## DISCUSSION

Chloride is the most abundant anion in the lumina of endosomes and lysosomes. Its concentration is ~70 mM in late endosomes and ~100 mM in lysosomes (*24*, *25*). These chloride ions play a crucial role in organellar physiology and function. Influx of Cl^−^ provides counterions to compensate H^+^ pumping and thereby facilitates the acidification of these organelles (*23*, *28*). Luminal Cl^−^ ions also regulate Ca^2+^ release from endosomes and lysosomes (*24*, *29*). The volume of endolysosomal lumens are much smaller than the volume of the cytoplasm, therefore even slight changes in Cl^−^ conductance of endolysosomal membrane could have a great influence on luminal Cl^−^ concentration. Endolysosomal Cl^−^ conductance is mediated by transporters and ion channels, including cystic fibrosis transmembrane conductance regulator (CFTR) (*30*), proton-activated Cl^−^ channel (*31*), and the CLC family members (*32*). Defects in these proteins lead to lysosomal dysfunction and storage disorders. In addition to these widely studied chloride channels, a lysosomal Cl^−^ current with distinctive properties has also been found, indicating the existence of other chloride transportation mechanisms (*33*). Here, we identified a novel endolysosomal Cl^−^-conducting protein, CLN7, which is a disease-associated protein underlying vLINCL. We found that KO of CLN7 eliminated most of the endogenous endolysosomal Cl^−^ currents in HEK293T cells and primary cultured macrophages in vitro (Figs. 5, B and C, and 7, D to F), indicating a crucial role of CLN7 in regulating Cl^−^ homeostasis. CLN7 has no sequence similarity to other known Cl^−^ channels. We have thus found a new family of Cl^−^ channel with a totally new structure.

CLN7 has long been considered to be a putative transporter. Unlike the clear differences between cation channels and transporters, it is difficult to distinguish between anion channels and transporters. For example, CFTR belongs to the ATP-binding cassette transporter family, but it is actually a chloride channel (*34*). The CLC family encompasses both Cl^−^/H^+^ antiporters and chloride channels (*35*). In our present study, we elucidated multiple lines of evidence, indicating that CLN7 acts more like a chloride channel. First, we found that the organellar Cl^−^ conductance mediated by CLN7 was extremely high, which would be difficult to achieve via transporters. In CLN7-overexpressing endolysosomes, we measured currents that exceeded 1 nA. Even endogenous CLN7 currents can reach hundreds of picoamperes, surpassing that of many other endogenous ion channel currents. Second, in our present study, we revealed that the characteristics of CLN7 currents were more like those of ion channels than they were of transporters. Our data showed that CLN7 mediated outward-rectifying currents, which is similar to the properties of ion channel currents of CLC-3 and CLC-7 (*36*, *37*). In addition, like most other chloride channels, CLN7 conducts other halide anions. Third, we demonstrated that CLN7 currents were sensitive to multiple chloride channel blockers. Fourth, unlike many transporters, we found that CLN7 did not rely on other ions or ATP to conduct Cl^−^ currents. Fifth, mutations of the amino acids in the predicted pore region of CLN7 largely reduced the Cl^−^ currents (fig. S8). Last, the pathogenic mutations E336Q and A157 change the selectivity of CLN7 to different halide ions (fig. S13), which strongly supports that CLN7 is as an ion channel.

Endosomes and lysosomes are acidic organelles. Low luminal pH values are required to maintain their functions, especially for lysosomes. Changes in lysosomal pH affect lysosomal enzymatic activity, as well as the lysosomal membrane potential, lysosomal trafficking, mammalian target of rapamycin signaling, and interactions with other organelles (*38*, *39*). The accumulation of H^+^ in lysosomes mainly depends on the action of vacuolar-type ATPases (*40*), but other lysosomal membrane proteins (e.g., lysosomal ion channels) also have a great effect on lysosomal pH. For example, in mucolipidosis type IV cells with TRPML1 mutations, lysosomal pH is increased to 5.2 (*41*). In transmembrane protein 175 (TMEM175) or two-pore channel 1 (TPC1)/TPC2 KO cells, although the lysosomal pH remains unchanged under normal conditions, it is increased significantly after starvation (*42*, *43*).

In addition, many CLN gene defects cause changes in lysosomal pH (*44*). However, unlike most CLN defects that induce an increase in lysosomal pH, we detected a decrease in lysosomal pH in CLN7-KO cells in our present study (Fig. 5, N to Q). This finding indicates that the activity of CLN7 maintains a higher pH value or leads to an increase in lysosomal pH. Furthermore, this finding is theoretically reasonable because CLN7 mediates an outward-rectifying Cl^−^ current, which means that it will transport Cl^−^ from the lysosomal lumen into the cytoplasm. This will result in a reduction in luminal counterions and that will concomitantly inhibit the transport of H^+^ into lysosomes. This hypothesis has been confirmed experimentally; Park and colleagues (*45*) have reported that two synthetic chloride transporters reduce the concentration of chloride in lysosomes and cause a rise in lysosomal pH.

In our present study, we revealed that the activity of CLN7 is also regulated by pH. A decrease in pH on the luminal side significantly increased CLN7 currents (Fig. 3, E to H). The ability of CLN7 to sense and regulate lysosomal pH makes it an ideal negative-feedback regulator. When lysosomal pH decreases, CLN7 is activated. The activation of CLN7 will increase the lysosomal pH, thereby stabilizing the pH within a specific range. A previous study reported that the lysosomal sodium channel, TPC1, is another feedback regulator of lysosomal pH. However, TPC1 acts in the opposite manner to that of CLN7. An increase in lysosomal pH activates TPC1, which subsequently promotes a decrease in lysosomal pH (*33*). Here, we also found that overexpression of TPC1 decreased the lysosomal pH, while knockdown of TPC1 increased the lysosomal pH (fig. S18). Therefore, TPC1 and CLN7 constitute a two-way feedback regulatory mechanism, which together control the lysosomal lumen to be maintained at approximately a pH of 4.5 (fig. S19).

In addition to lysosomal pH, we found that CLN7 also regulates lysosomal calcium content. Lysosomes are important intracellular calcium stores. Overexpression of CLN7 promotes lysosomal calcium release through a TRPML1-dependent way (Fig. 5, F and G, and fig. S19). Calcium ions are an important second messenger and participate in a variety of intracellular signaling pathways, so this lysosomal calcium release may have a wide range of effects. One of the consequences we found is the activation of CaM and the resulting lysosomal fusion and enlargement. When we overexpress four known CLN7 pathogenic mutations—namely, E336Q, A157P, T294K, and R35stop—we found that the mutations that caused more severe symptoms failed to induce endolysosomal enlargement and increases in Cl^−^ currents. Hence, this overexpression-induced enlargement of endolysosomes may result from the increase of Cl^−^ conductance. A previous study has also reported that overexpression of another chloride channel, CLC-3, induces large, acidic vesicular structures in Chinese hamster ovary (CHO-K1) or human hepatoma (Huh-7) cells (*46*). CLN7 exhibits markedly similar electrophysiological characteristics to those of CLC-3 (*37*). Both proteins exhibit outward rectification and low-pH activation. Their current activation curves are also similar.

Lysosomal membranes have higher permeabilities to I^−^ than they do to Cl^−^ (*47*). Consistent with this characteristic, CLN7 is also more permeable to I^−^ (Fig. 3, A to D). In addition to patch-clamp recordings, we also used an iodide-sensitive YFP variant to verify the permeability of CLN7 to I^−^ in the present study (fig. S9, A and B). These findings indicate that CLN7 is involved in the transport of iodide ions. Since CLN7 mainly transports anions from the lysosomal cavity to the cytoplasm, KO of CLN7 may cause accumulation of I^−^ in lysosomes, which may affect lysosomal enzymatic activities (*48*). Therefore, changes in I^−^ balance may be related to the pathogenesis of CLN7-related diseases.

There are different chloride channels and transporters located on the lysosomes. Because of differences in their channel characteristics, tissue distributions, cellular localizations, and intracellular regulatory mechanisms, their mutations can cause distinct types of diseases. For instance, mutations in the gene encoding CLC-7 have been found to be related to osteopetrosis (*49*). Loss of CLC-3 leads to a variety of defects including hippocampal degeneration, impaired insulin secretion, and cardiovascular disease (*50*–*52*). Defects in CFTR are known to cause cystic fibrosis, resulting in lung disease, pancreatic insufficiency, and other lethal diseases (*53*). CLN7 has also been reported to be involved in vLINCL. However, how CLN7 deficiency induces this disorder has remained unclear. In the present study, we demonstrated that CLN7 regulates lysosomal Cl^−^ conductance, lysosomal membrane potential, and luminal pH. The luminal pH is a key factor affecting lysosomal enzyme activity. Most lysosomal enzymes work best at a low pH. On the contrary, some enzymes require relatively high pH values in certain situations (*54*). In the present study, in CLN7-KO and knockdown HEK293T cells, we detected reduced lysosomal pH, which is likely to cause a decrease in the activities of such enzymes. In addition to lysosomal pH, CLN7-mediated regulation of the lysosomal membrane potential may also affect lysosomal function by affecting other voltage-sensitive ion channels expressed on the lysosomal membrane. The voltage-sensitive two-pore channel, TPC1, is one of the nutrient sensors in lysosomes and participates in the transport of amino acids (*55*). Therefore, defects in CLN7 may also disturb lysosomal nutrient homeostasis. Another voltage-gating channel, TRPML1, is a major Ca^2+^-conductive protein of lysosomes (*56*). Changes in membrane potential affect the release of lysosomal Ca^2+^ and subsequent signal transduction. Here, we show that overexpression of CLN7 promotes TRPML1-mediated lysosomal calcium release, which, in turn, activates CaM and promotes lysosomal fusion (Figs. 1, C to J, and 5, D to G; and fig. S19). The effect of CLN7 on TRPML1 may be achieved by affecting the lysosomal membrane potential.

Although it is unclear how CLN7 deficiency induces neurodegeneration, there are numerous studies that have revealed a regulatory role of CLN7 in neurodevelopment and synaptic function. CLN7 has been shown to be enriched at postsynaptic regions of *Drosophila* neuromuscular junctions (*57*). Furthermore, loss of *CLN7* has been demonstrated to impair synaptic development and consequently induce synaptic dysfunction (*58*). Another endolysosomal chloride channels, CLC-3, is also involved in the regulation of neurodevelopment and synaptic transmission. CLC-3 has been found to be expressed in synaptic vesicles and postsynaptic membranes. Presynaptical CLC-3 facilitates vesicular acidification, while postsynaptic CLC-3 modulates excitatory postsynaptic potentials. Moreover, CLC-3 KO causes severe hippocampal and retinal degeneration in mice (*50*). These studies highlight the importance of chloride homeostasis in the functioning of the nervous system.

In summary, we have identified CLN7 as a novel endolysosomal chloride channel that regulates lysosomal chloride homeostasis, lysosomal pH, and lysosomal membrane potential. KO of CLN7 in mice mimicked the pathological features of human vLINCL, including autofluorescent lipofuscin deposition and accumulation of ATP synthase subunit C. Furthermore, we established that these symptoms were due to impaired chloride transport in endolysosomes. Together, our findings suggest that development of specific drugs to restore the function of mutant CLN7 or to regulate other similar lysosomal chloride ion channels/transporters to compensate for the lack of CLN7-mediated chloride transport is expected to be a promising therapeutic strategy for mitigating CLN7-related diseases.

## MATERIALS AND METHODS

### Animals

All animal experimental procedures were approved by the Animal Care and Use Committee of the University of Science and Technology of China. A CLN7-KO mouse model was generated by the Laboratory Animal Center in the School of Life Sciences at the University of Science and Technology of China. Zygotes derived from WT C57BL/6N mice were coinjected with Cas9 mRNA and two sgRNAs targeting the fourth and sixth exons to introduce fragment deletion into the *CLN7* gene. A founder strain with a 3148-bp deletion in the *CLN7* gene locus was used for breeding. Heterozygous mice were backcrossed to C57BL/6N for more than five generations before being used to generate homozygous KOs.

### Cell culture

All cells were cultured at 37°C in a humidified incubator supplied with 5% CO_2_. HeLa and HEK293T cells were maintained in Dulbecco’s modified eagle medium (DMEM; Gibco) supplemented with 10% fetal bovine serum (FBS; Biological Industries), 1× glutagro (Corning), and 1× penicillin/streptomycin (Biosharp). SH-SY5Y cells were maintained in DMEM/F12 supplemented with 20% FBS, 1× glutagro, and 1× penicillin/streptomycin. MEFs were cultured from embryonic day 14.5 (E14.5) embryos. The embryos were harvested from sacrificed pregnant mice, soaked in 70% ethanol, and washed with sterile phosphate-buffered saline (PBS). The heads, tails, limbs, and internal organs were removed under a dissecting microscope. Then, the rest of the other parts of the bodies were minced into 1-mm pieces and digested with 0.25% trypsin-EDTA for 30 min at 37°C. MEF cells were dissociated by pipetting up and down and were then seeded into 10-cm dishes with 12 ml of DMEM supplemented with 10% FBS, 1× glutagro, and 1× penicillin/streptomycin. Mouse peritoneal macrophages were isolated and cultured following previously described protocols (*59*) but with a slight modification. Briefly, adult mice were sacrificed and injected with 10 ml of ice-cold PBS into the peritoneal cavity. After gently massaging the peritoneum, the intraperitoneal PBS was collected and centrifuged at 400*g* for 10 min at 4°C. Pelleted cells were resuspended with DMEM medium supplemented with 20% FBS and 1× penicillin/streptomycin, after which the cells were seeded onto poly-l-lysine–coated coverslips. At 2 hours before whole-endolysosomal electrophysiological recordings, the cells were treated with 1 μM vacuolin-1 to enlarge the endolysosomes.

### Electrophysiology

Electrophysiological recordings were performed with a Multiclamp 700B amplifier and a Digidata 1550B data acquisition system (Molecular Devices). PClamp, Clampfit (Molecular Devices), and OriginPro (OriginLab) software were used to record and analyze data. For whole-cell recordings presented in Fig. 4 and fig. S9, the bath solution contained 150 mM HCl, 10 mM Hepes, and 10 mM MES (pH adjusted to 4.6 with NMDG), while the pipette solution contained 150 mM HCl and 20 mM Hepes (pH adjusted to 7.2 with NMDG). Patch-clamp recordings were achieved using enlarged organelles following previous described methods (*29*, *42*, *60*). Endolysosomes of HEK293T cells were enlarged by overnight treatment with 1 μM vacuolin-1 and were dissected out of cells with glass patch-clamp pipettes before recordings. Unless otherwise indicated, the bath solution used in endolysosomal voltage-clamp recordings contained 149 mM methanesulfonic acid, 1 mM HCl, and 20 mM Hepes (pH adjusted to 7.2 with NMDG), while the pipette solution contained 150 mM HCl, 10 mM Hepes, and 10 mM MES (pH adjusted to 4.6 with NMDG). For current-clamp recordings presented in Fig. 5 (J to M), the bath solution contained 140 mM KCl, 5 mM NaCl, 2 mM MgCl_2_, 0.5 mM CaCl_2_, 1 mM EGTA, and 10 mM Hepes (pH adjusted to 7.2 with KOH). The Cl^−^ concentration was adjusted by partial substitution of the bath solution with an appropriate amount of low chloride solution containing 140 mM K-gluconate, 5 mM Na-gluconate, 2 mM Mg(OH)_2_, 0.5 mM CaCl_2_, 1 mM EGTA, and 10 mM Hepes (pH adjusted to 7.2 with KOH). The pipette solution contained 145mM NaCl, 5 mM KOH, 1 mM MgCl_2_, 1.5 mM CaCl_2_, 10 mM glucose, 10 mM Hepes, and 10 mM MES (pH adjusted to 4.6 with methanesulfonic acid). The liquid junction potentials were corrected online for the voltage-clamp recordings and were corrected after experiments for the current-clamp recordings. An agar salt bridge was used with the reference electrode.

For recordings presented in Figs. 2 (A to C) and 5 (H and I), the bath solution contained 140 mM K-gluconate, 4 mM NaCl, 1 mM EGTA, 2 mM MgCl_2_, 0.39 mM CaCl_2_, and 10 mM Hepes (pH adjusted to 7.2 with KOH); in contrast, the pipette solution contained 145 mM NaCl, 5 mM KCl, 2 mM CaCl_2_, 1 mM MgCl_2_, 10 mM glucose, 10 mM Hepes, and 10 mM MES (pH adjusted to 4.6 with NaOH). For recordings presented in Fig. 2G, the bath solution contained 150 mM HCl and 20 mM Hepes (pH adjusted to 7.2 with NMDG), while the pipette solution contained 150 mM HCl, 10 mM Hepes, and 10 mM MES (pH adjusted to 4.6 with NMDG). For recordings presented in Fig. 2I, the bath solution contained 140 mM methanesulfonic acid, 10 mM HCl, and 20 mM Hepes (pH adjusted to 7.2 with NMDG), whereas the pipette solution contained 150 mM HCl, 10 mM Hepes, and 10 mM MES (pH adjusted to 4.6 with NMDG). For recordings presented in Fig. 3 (A to D) and fig. S13, the pipette solution contained 150 mM NaCl/NaF/NaI, 10 mM Hepes, and 10 mM MES (pH adjusted to 4.6 with NMDG). For recordings presented in fig. S6A (left), the pipette solution contained 154 mM NMDG-Cl, 10 mM Hepes, and 10 mM MES (pH adjusted to 4.6 with NMDG). For recordings presented in fig. S6A (middle), the pipette solution contained 75 mM NaCl, 75 mM KCl, 1 mM CaCl_2_, 1 mM MgCl_2_, 10 mM Hepes, and 10 mM MES (pH adjusted to 4.6 with NMDG).

The inhibition curves presented in Fig. 3 (L, N, and P) were fitted with the equation

$$I/I_{0}=1/[1+(X/{\text{IC}}_{50})h]$$

where *I* and *I*_0_ are the current amplitudes obtained in the presence and absence of blockers, respectively, *X* is the blocker concentration, IC_50_ is the concentration for half-maximal inhibition, and *h* is the Hill coefficient.

The voltage dependence of activation (Fig. 2J and fig. S12, C and F) was studied using a 500-ms voltage-step protocol (0 to +190 mV, 10 mV step; Vh = −70 mV). The activation curves were fitted using the Boltzmann equation, as follows

$$G/G_{\text{max}}=F-F/(1+\text{exp}\;[(V_{1/2}-V)/\kappa ])$$

where *G* is the conductance, *F* is the final value of *G/G*_max_ acquired by fitting, *V*_1/2_ is the half-maximum activation voltage, *V* is the testing voltage, and κ is the slope factor.

### cDNA constructs and transfections

The N-terminally EGFP-tagged hCLN7 constructs were gifts from B. Gasnier (Paris Descartes University) and were previously described (*14*). EGFP-tagged hCLN7 constructs were generated by inserting the cDNA into the Eco RI site of pEGFP-C2 or the Xho I and Kpn I sites of pEGFP-N1 vectors. In addition, mCherry-tagged CLN7 was generated by inserting the cDNA of hCLN7 into Eco RI and Xba I sites of the mCherry2-C1 vector. For nontagged CLN7, the cDNA was subcloned into Eco RI and Xba I sites of the pcDNA3.1(+) vector. CLN7 mutations were generated by PCR from pEGFP-C2-CLN7 and were confirmed by sequencing. EYFP-H148Q/I152L was generated by PCR from pEYFP-C1. The CLN7 homolog of *S. cerevisiae* (YCR023C) was synthesized with codon optimization and was subcloned into Xho I and Kpn I sites of pEGFP-C1. For Lamp2, the cDNA was subcloned into Xho I and Hind III sites of the mCherry2-N1 or pEGFP-N1 vector. For mCLN7, the cDNA was subcloned into Xho I and Kpn I sites of the pEGFP-C1 vector. EGFP-tagged human TRPML1 construct was generated by inserting the cDNA into the Kpn I and Sma I sites of pEGFP-C1 vector. The CaM_1,2,3,4_ mutant is a rat CaM with the first aspartic acid residue of each of the four EF hands mutated to alanine and is cloned into the Kpn I and Xba I sites of vector pcDNA3 (a gift from D. Yue). For hTPC1, the cDNA was subcloned into Nhe I and Eco RI sites of the pcDNA3.1(+) vector. Transient expression of DNA constructs was performed with Lipofectamine 2000 (Invitrogen) according to the manufacturer’s protocol.

For lentiviral expression constructs, the cDNA of mCLN7 was PCR-amplified and subcloned into Eco RI and Bam HI sites of pSin-EF2-3xFlag-puro. Lentiviral particles were produced by cotransfection of the lentiviral expression constructs with the packing plasmid, psPAX2, and envelope plasmid, pMD2.G, into HEK293T cells via LentiFit (HANBIO) transfection reagent. Viruses were collected at 2 days after transfection and were added to target cells in the presence of polybrene (8 μg/ml; Sigma-Aldrich).

### CRISPR KO of CLN7 in HEK293T cells

Using CRISPR-Cas9 technology to knock out CLN7 in HEK293T cells, the targeted hCLN7 sequence, namely, CCTGCGGAACGAAAGTGAAC[AGG] (with AGG serving as the PAM motif), was targeted using the plentiCRISPR-V2 vector. Lentiviruses were generated by cotransfecting HEK293T cells with plentiCRISPR-V2, lentiviral packing (psPAX2), and envelope (pMD2.G) plasmids via Lipofectamine 3000 (Invitrogen) transfection reagent. At 48 hours after transfection, the culture medium containing lentiviral particles was collected and added to fresh HEK293T cells cultured in antibiotic-free medium containing polybrene (8 μg/ml). Starting at 24 hours after infection, the cells were selected with puromycin (3 μg/ml) for 48 hours. Single-cell clones were established using a limiting dilution method and were then sequenced to confirm KO of CLN7.

### shRNA knockdown

The shRNA of CLN7 and TPC1 were gifts from G. Shan (University of Science and Technology of China). The targeted sequence for hCLN7 was GCTGGGTTATTGCTTCATATA. To generate shRNA-encoding plasmids, oligo pairs [5′ phosphorylated; for hCLN7, CCGGGCTGGGTTATTGCTTCATATACTCGAGTATATGAAGCAATAACCCAGCTTTTTTG (forward) and AATTCAAAAAAGCTGGGTTATTGCTTCATATACTCGAGTATATGAAGCAATAACCCAGC (reverse)] were annealed and ligated into the Age I/Eco RI sites of pLKO.1. The targeted sequence for hTPC1 was GCCTACCTCTTTGCACACAAT. To generate shRNA-encoding plasmids, oligo pairs [5′ phosphorylated; for hTPC1, CCGGGCCTACCTCTTTGCACACAATCTCGAGATTGTGTGCAAAGAGGTAGGCTTTTTG (forward) and

AATTCAAAAAAGCCTACCTCTTTGCACACAATCTCGAGATTGTGTGCAAAGAGGTAGGC (reverse)] were annealed and ligated into the Age I/Eco RI sites of pLKO.1. Scramble shRNA was a gift from H. Zhang (University of Science and Technology of China). The procedures for the production of lentiviruses and transfection and selection of puromycin-resistant cells were the same as those used when knocking out CLN7 in HEK293T cells (mentioned above). At 4 days after viral infections, knockdown of CLN7 was verified by Western blotting of CLN7 protein.

### Real-time quantitative polymerase chain reaction

Total RNA was extracted from WT and CLN7-KO MEFs using RNA Isolater Total RNA Extraction Reagent (Vazyme) according to the manufacturer’s instructions. Purified RNA was quantified using NanoDrop 2000 (Thermo Fisher Scientific). The cDNA was synthesized by reverse transcription using HiScript II Q RT SuperMix (Vazyme). RT-qPCR was performed on LightCycler 96 (Roche) using SYBR Green Master (Vazyme). The following primers were used: 5′-TGTTGCCGTTGTCCGATCAT-3′ (forward) and 5′-GATGTCCCACGTCACACCTT-3′ (reverse) for CLN7 and 5′-TGATGGGTGTGAACCACGAG-3′ (forward) and 5′-GCCCTTCCACAATGCCAAAG-3′ (reverse) for Gapdh. The 2^-ΔΔCt^ method was used to calculate the relative mRNA level of CLN7.

### Protein chemistry

For the Western blots used in Fig. 7B and figs. S10, S11, and S18, the cells were harvested in Nonidet P-40 lysis buffer (Biosharp) supplemented with protease inhibitor (Thermo Fisher Scientific) and incubated on ice for 30 min. After centrifugation at 18,500*g* for 10 min, the supernatants were collected and heated for 15 min at 72°C. Samples were separated on a 10% SDS–polyacrylamide gel electrophoresis (SDS-PAGE) gel using a vertical electrophoresis system and transferred onto polyvinylidene difluoride (PVDF) membranes using a transblot electrophoretic transfer system (Cavoy). Membranes were then blocked for 2 hours in tris-buffered saline (TBS) buffer with 5% skimmed milk and 0.1% Tween-20. A rabbit polyclonal CLN7 antibody (Atlas Antibodies, catalog no. HPA044802), a rabbit polyclonal TPC1 antibody (Proteintech, catalog no. 23758-1-AP), and glyceraldehyde-3-phosphate dehydrogenase (GAPDH) antibody (Proteintech, catalog no. 66305-1-zg) were separately used at dilutions of 1:250, 1:500, and 1:2000, respectively, for Western blot analysis.

Mouse cerebellums and livers were used to test the accumulation of mitochondrial ATP synthase subunit C (Fig. 7K). Six-month-old WT and CLN7-KO mice were deeply anesthetized with isoflurane and sacrificed by cervical dislocation. The cerebellums and livers were quickly dissected and separately homogenized in 0.25 M sucrose buffer containing 50 mM tris (pH 7.4), 1 mM EDTA, and protease inhibitor. The homogenates were centrifuged at 500*g* for 10 min to obtain the resultant supernatants, and Triton X-100 was then added to yield a 1% concentration. The supernatants were incubated on ice for 30 min and were then centrifuged at 13,000*g* for 15 min. The soluble fractions were collected and kept on ice. The insoluble fractions were harvested in 50 mM of tris buffer (pH 7.5) containing 2% SDS and protease inhibitor to lyse these fractions on ice for an additional 30 min. Then, the samples were denatured at 100°C for 5 min, separated on a 15% SDS-PAGE gel, and transferred onto PVDF membranes. Membranes were blocked for 2 hours in TBS buffer with 5% skimmed milk and 0.1% Tween-20. An ATP synthase C antibody (Abcam, catalog no. ab181243) and GAPDH antibody (Proteintech, catalog no. 66305-1-zg) were used at 1:2000 dilutions at 4°C overnight. A horseradish peroxidase–conjugated secondary antibody (1:2000) from a Western blotting detection kit (Advansta) was used for protein detection.

### Preparation of brain slices and immunostaining

Nine-month old CLN7-KO and WT mice were deeply anesthetized with isoflurane and then perfused with chilled PBS, followed by 4% paraformaldehyde in PBS. Then, the brains were removed and transferred into a series of sucrose solutions (10, 20, and 30% sucrose) until they sank at 4°C. Subsequently, 40-μm-thick coronal brain sections were obtained using a freezing cryostat (Leica, CM1860). The brain slices for autofluorescence detection (fig. S16, B and C) were mounted on microscope slides with fluorescent mounting medium for subsequent confocal imaging. The hippocampal slices for CLN7 immunostaining (fig. S16A) were washed in PBS and blocked overnight at 4°C in PBS containing 6% goat serum (v/v), 1% bovine serum albumin (BSA) (w/v), and 0.2% Triton X-100. Then, the slices were incubated with anti-MFSD8 antibodies (1:100: Atlas Antibodies, HPA044802) at 4°C for 3 days in PBS containing 3% goat serum (v/v), 0.2% Triton X-100, and 0.5% BSA (w/v). After washing three times with PBS (10 min each time), brain sections were incubated with the secondary antibody, fluorescein isothiocyanate–conjugated goat anti-rabbit immunoglobulin G (IgG) (H+L) (1:200; Elabscience, E-AB-1055), for fluorescent detection.

### Confocal imaging

Images were taken using a confocal system consisting of a Nikon Eclipse Ti inverted microscope, a CSU-X1 Spinning Disk Unit (Yokogawa), a DU-897 U electron-multiplying charge-coupled device camera (Andor), a laser-controlling module (Andor), and iQ3 imaging software (Andor). For protein localization, HEK293T, HeLa, and SH-SY5Y cells that were transfected for at least 12 hours were trypsinized and seeded in culture dishes with thin glass bottoms coated with poly-l-lysine. For lysosomal calcium imaging (Fig. 5, D and E), HEK293T cells were incubated with OG488 BAPTA-1 dextran (10 μg/ml; Thermo Fisher Scientific) for 14 hours at 37°C and then cultured in medium without OG488 BAPTA-1 dextran for another 2 hours. For the data shown in Fig. 5E, the cells in each group were from the same passage. For WT cells, *n* = 7837 lysosomes from 235 cells. For KO cells, *n* = 5497 lysosomes from 254 cells. For WT + hCLN7 cells, *n* = 6157 lysosomes from 210 cells. Images were acquired with a 100× oil-immersion lens at 488 nm for EGFP and OG488 BAPTA-1 dextran and at 594 nm for mCherry, LysoTracker Red, and DiD. For autofluorescent detection in Fig. 7I and fig. S16 (B and C), images were acquired at an excitation wavelength of 488 nm with a 60× oil-immersion and 10× objective lens, respectively. ImageJ was used to analyze fluorescent intensities.

### Calcium imaging

Cells grown on poly-l-lysine–coated coverslips were loaded with 2 μM Fura-2 AM and 0.02% pluronic F-127 for 30 min at 37°C in isotonic solution containing 135 mM NaCl, 5.4 mM KCl, 1.8 mM CaCl_2_, 0.9 mM MgCl_2_,10 mM glucose, and 10 mM Hepes (pH 7.4 by NaOH). The same solution but without CaCl_2_ was used as imaging buffer. Cells were then washed twice with imaging buffer and transferred into the imaging chamber. GPN (600 μM) was applied to the cells to induce Ca^2+^ release from lysosomes. TG (2 μM) was applied to the cells to induce Ca^2+^ release from endoplasmic reticulum. Calcium transients were captured using a calcium imaging system consisting of a DG-5 wavelength switcher (Sutter Instrument), an ORCA-Flash4.0 LT+ complementary metal oxide-semiconductor (CMOS) camera (Hamamatsu), and a Ti2 microscope (Nikon). Data were collected and analyzed using MetaFluor software (Molecular Devices). Ratiometric measurements were performed by switching the excitation wavelength from 340 to 380 nm and quantifying emission at 510 ± 40 nm.

### Lysosomal pH imaging

LysoSensor was used to monitor changes in lysosomal pH under different treatments. HEK293T, HeLa, and SH-SY5Y cells were seeded in culture dishes with thin glass bottoms coated with poly-l-lysine. Before imaging, cells were loaded with 1 μM LysoSensor Green (Invitrogen) in culture medium for 30 min at 37°C. Cells were then washed twice with PBS, transferred into fresh culture medium, and imaged immediately. Fluorescence was excited at 445 nm and filtered with a 525 ± 25 nm emission filter, and images were acquired with a spinning-disk confocal microscope with a 100× oil-immersion lens. For the data shown in Fig. 5O, the cells in each group were from the same passage. For WT cells, *n* = 1124 lysosomes from 64 cells. For KO cells, *n* = 1896 lysosomes from 84 cells. For KO + mCLN7 cells, *n* = 1560 lysosomes from 68 cells. The cells in each group were from the same passage.

For quantitative measurements of lysosomal pH, OG488–conjugated dextran was used following a previously described method (*43*). Briefly, cells were seeded into 35-mm culture dishes with thin glass bottoms and incubated with OG488–conjugated dextran (250 μg/ml; Invitrogen) overnight. Before imaging, cells were washed once with imaging buffer. The imaging solution contained 140 mM NaCl, 3 mM KCl, 2 mM K_2_HPO_4_, 1 mM CaCl_2_, 1 mM MgSO_4_, and 5 mM Hepes (pH7.4). Images were acquired at excitation wavelengths of 488 and 445 nm. The emission was filtered at 525 ± 25 nm. The calibration solutions (5 mM NaCl, 115 mM KCl, 1.2 mM MgSO_4_, 10 mM glucose, and 25 mM Hepes, supplemented with 10 μM nigericin and monensin) with a pH ranging from 3.5 to 7.0 were used as pH standard solutions. pH calibration was performed at the end of imaging for each coverslip. Under different pH conditions, the ratio (488 nm/445 nm) of the obtained fluorescent intensity was fitted to a Boltzmann sigmoidal curve to acquire the pH standard curve. The pH values of lysosomes were calculated by fitting their fluorescent ratios to the pH standard curve.

### Iodide imaging

EYFP-H148Q/I152L was used to monitor changes in intracellular iodide concentrations. The fluorescent intensity of this YFP variant decreases with increasing iodide concentrations. HEK293T cells were cotransfected with EYFP-H148Q/I152L and the indicated plasmid at 36 hours before imaging and were plated onto poly-l-lysine–coated coverslips. Cells were washed with PBS and monitored using an imaging system consisting of a DG-5 wavelength switcher (Sutter Instrument), an ORCA-Flash4.0 LT+ CMOS camera (Hamamatsu), and a Ti2 microscope (Nikon). The imaging buffer contained 140 mM NaCl, 3 mM KCl, 2 mM K_2_HPO_4_, 1 mM CaCl_2_, 1 mM MgSO_4_, and 5 mM Hepes (pH of 7.4). The fluorescent intensities were quantified with MetaFluor software (Molecular Devices).

### Flow cytometry

Lipid peroxidation and lysosomal degradation ability were analyzed by flow cytometry. For lipid peroxidation analysis, cells were stained with 2 μM C11 BODIPY for 60 min at 37°C. For degradation ability analysis, cells were loaded with DQ Green BSA (50 μg/ml) for 30 min at 37°C and then incubated with fresh medium without DQ Green BSA for another 30 min. Cells were then washed with ice-cold PBS and resuspended in PBS for flow cytometry analysis. Before testing, 4′,6-diamidino-2-phenylindole (DAPI; 1 μg/ml) was added to mark dead cells. The signals of BODIPY and DQ Green were analyzed on the 525/40 bandpass filter channel, while DAPI signal was analyzed on the 450/45 bandpass filter channel of a CytoFLEX cytometer (Beckman Coulter).

### Retinal immunostaining

Dissected retinae were fixed with 4% paraformaldehyde in PBS for 2 hours at 4°C, cryoprotected in PBS containing 30% sucrose for several hours at 4°C, and were embedded in optimum cutting temperature compound (Sakura Finetek) on dry ice. Cryosections (18 μm) were cut on a cryostat (Leica, CM3050S). Sections were stained with DAPI, anti-opsin (1:500; Sigma-Aldrich, catalog no. O4886), anti-medium-wavelength opsin (1:500; Millipore, catalog no. AB5407), or anti-short-wavelength opsin (1:500; Millipore, catalog no. AB5405). Sections were incubated with the secondary antibodies, Alexa Fluor 488 goat anti-rabbit IgG (H+L) (1:500; Thermo Fisher Scientific, catalog no. A11034) and Alexa Fluor 488 donkey anti-mouse IgG (H+L) (1:500; Thermo Fisher, catalog no. A21202) for fluorescent detection.

### Visual water maze test

The task is to assess the ability of 6-month-old WT and CLN7-KO mice to associate a specific visual stimulus (sine-wave gratings) with escape from water. The training/testing was conducted in a trapezoidal-shaped pool filled with 21° ± 1°C tap water made opaque with titanium dioxide. At the end of the goal arms, there are two computer-controlled monitors placed side by side, one of which displays vertically oriented sine-wave gratings and the other displays homogeneous gray. The patterns displayed on the two monitors will switch randomly for each trial. The same pattern cannot appear three times continuously on the same side. A hidden platform for escape is always placed at the end of the goal arm that displays the sine-wave gratings (fig. S17E).

For the training phase, mice are released into the swim tank facing the screen, near the hidden platform. Once the platform is found, they will be allowed to stay on it for 30 s to observe surroundings. Then, the mice will be released in the tank away from the platform gradually until they could find it from the end of starting arm. After all WT mice make correct choice (find the correct direction and swim onto the escape platform within 60 s) in three continuous trials, we will proceed to the subsequent testing trials.

For the testing trials, mice are released into the tank at the starting arm and allowed to swim freely to find the platform. Each mouse undergoes 18 testing trials. Once the mice pass the choice line correctly and reach the platform within 60 s, it will be judged as success. The success rate and time taken to the platform (escape latency) are recorded and the values of 18 trials are averaged. When the animal fails to complete the task within 60 s, a maximum escape latency of 60 s is assigned.

### Quantification and statistical analysis

Data were analyzed using ImageJ, Clampfit (Molecular Devices), Origin (OriginLab), and Excel (Microsoft). Numeric data are reported as the means ± SEM. Statistical significance was calculated with two-sided Student’s *t* tests or analysis of variance (ANOVA) with post hoc tests. A *P* < 0.05 was considered to be statistically significant and is denoted as follows in the figures: *P < 0.05, **P < 0.01, and ***P < 0.001.

## Notes

View/request a protocol for this paper from *Bio-protocol*.
