# Supplementary material for: CLN7 is an organellar chloride channel regulating lysosomal function
Source: Sci Adv. 2021 Dec 15;7(51):eabj9608. doi: 10.1126/sciadv.abj9608 (PMC8673761; doi:10.1126/sciadv.abj9608)
Supplement: Supplementary file 1 — Figs. S1 to S19 [file sciadv.abj9608_sm.pdf]

Science Advances
AAAS

Supplementary Materials for

CLN7 is an organellar chloride channel regulating lysosomal function

Yayu Wang, Wenping Zeng, Bingqian Lin, Yichuan Yao, Canjun Li, Wenqi Hu, Haotian Wu, Jiamin Huang, Mei Zhang, Tian Xue, Dejian Ren, Lili Qu*, Chunlei Cang*

*Corresponding author. Email: lilisqu@ustc.edu.cn (L.Q.); ccang@ustc.edu.cn (C.C.)

Published 15 December 2021, *Sci. Adv.* **7**, eabj9608 (2021)
DOI: 10.1126/sciadv.abj9608

This PDF file includes:

Figs. S1 to S19

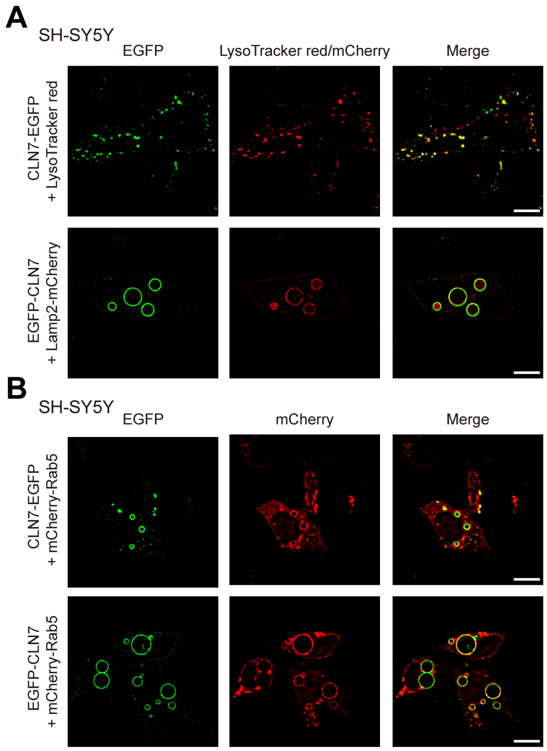

Fig. S1. CLN7 localizes to and enlarges lysosomes and endosomes in SH-SY5Y cells. (**A**) Co-localization of C-terminally EGFP-tagged CLN7 with LysoTracker red (upper) or N-terminally EGFP-tagged CLN7 with Lamp2-mCherry (lower) in SH-SY5Y cells. (**B**) Co-localization of C-terminally (upper) or N-terminally (lower) EGFP-tagged CLN7 with mCherry-Rab5 in SH-SY5Y cells. Scale bars = 10 µm.

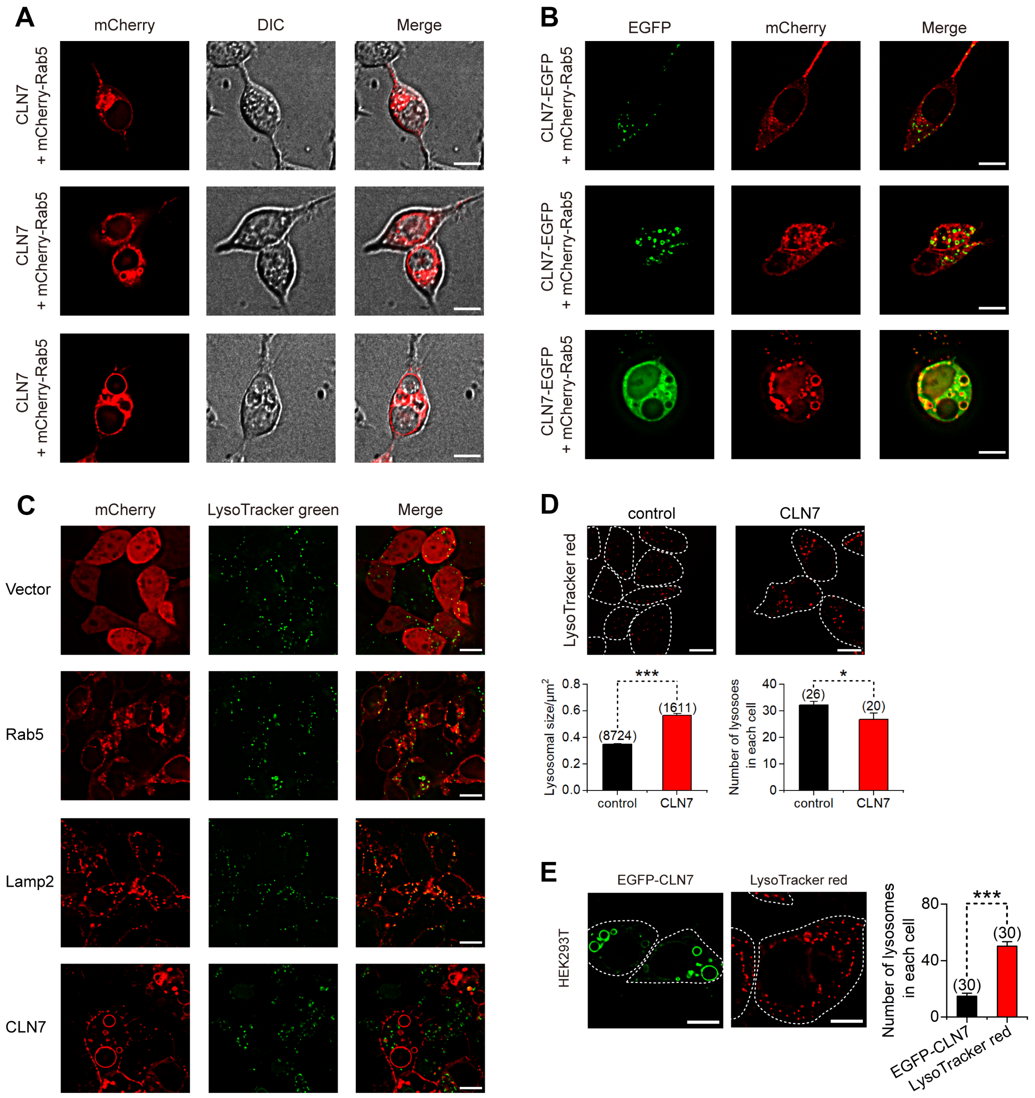

Fig. S2. Effects of non-tagged CLN7 and C-terminally EGFP-tagged CLN7 on endosomal enlargement in HEK293T cells

(**A** and **B**) Endosomal morphology in HEK293T cells transfected with non-tagged (**A**) or C-terminally EGFP-tagged CLN7 (**B**). Endosomes were labeled with mCherry-tagged Rab5. Slightly and moderately enlarged endosomes were observed in most cells (top and middle). Notably enlarged endosomes were observed in a small portion of cells (bottom). (**C**) Endolysosomal morphology in HEK293T cells expressing mCherry vector, mCherry-Rab5, Lamp2-mCherry or N-terminally mCherry-tagged CLN7. All cells were stained with LysoTracker green. Scale bars = 10 μm. (**D**) (Upper) Lysosome labeling with LysoTracker red in HEK293T cells transfected with mock vector (control) or non-tagged CLN7. (Lower) Averaged size of lysosomes and number of lysosomes in each cell represented in (**D** upper). For control cells, n = 8724 lysosomes from 262 cells on 1 passage. For CLN7-transfected cells, n = 1611 lysosomes from 63 cells on 1 passage. (**E**) (Left) Representative fluorescent images of HEK293T cells expressing EGFP-CLN7 or stained with LysoTracker red. (Right) Averaged numbers of lysosomes in cells represented in (**E** left). Data are presented as the mean ± SEM.

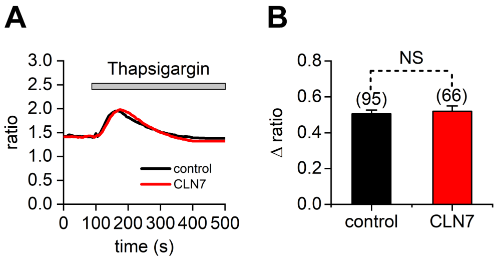

Fig. S3. Expression of CLN7 does not affect thapsigargin-induced endoplasmic reticulum calcium release.

(**A**) Representative Fura-2 fluorescence ratio (340/380) showing the effect of thapsigargin on intracellular [Ca^{2+}] in HEK293T cells transfected with empty vector (control) or non-tagged CLN7. (**B**) Thapsigargin-induced changes in Fura-2 fluorescence ratio (340/380) in cells with different transfection. Data are presented as the mean ± SEM.

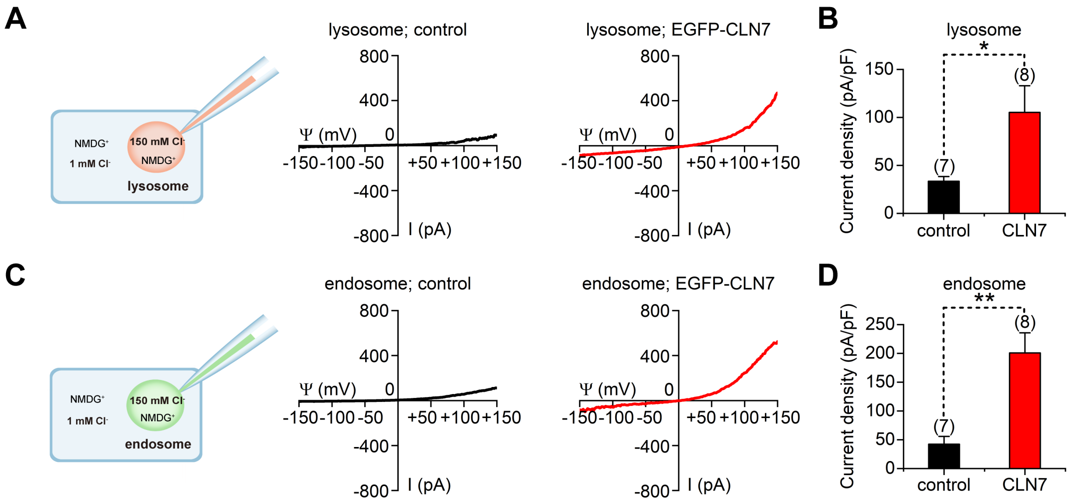

Fig. S4. CLN7 mediates Cl⁻ currents in lysosomes and endosomes.

(**A-D**) Cl⁻ currents recorded in lysosomes (**A** and **B**) and endosomes (**C** and **D**) from EGFP-CLN7-transfected HEK293T cells. Lysosomes were labeled with Lamp2-mCherry, while endosomes were labeled with mCherry-Rab5. Recording conditions and representative traces are in (**A**) and (**C**). Current densities (measured at +150 mV) are shown in (**B**) and (**D**). Data are presented as the mean ± SEM.

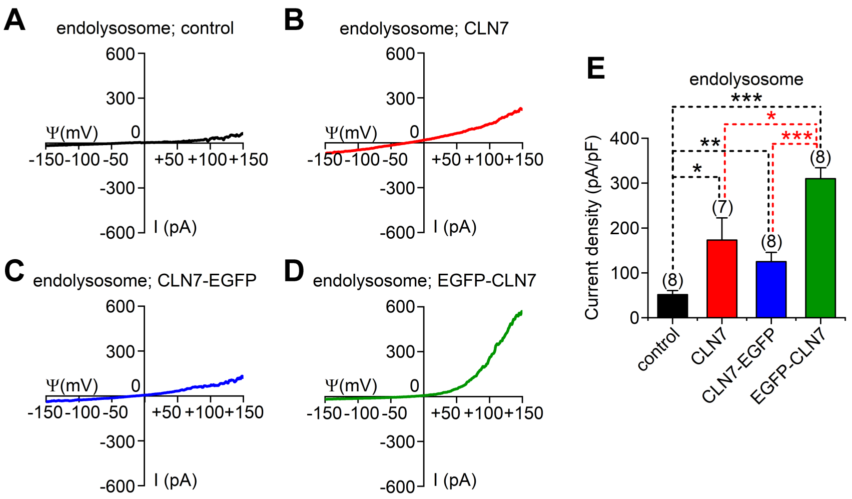

Fig. S5. CLN7 with different tag mediate Cl⁻ currents in endolysosomes.

(**A-D**) Whole endolysosomal currents recorded from HEK293T cells transfected with mock vector (control), CLN7, CLN7-EGFP, or EGFP-CLN7. (**E**) Current densities of the endolysosomes represented in (**A-D**). All recordings were performed under Cl^--based conditions with Cl^- as the major inorganic ion (1 mM of Cl^- in the bath solution and 150 mM of Cl^- in the pipette solution). Current densities were measured at +150 mV. Data are presented as the mean ± SEM.

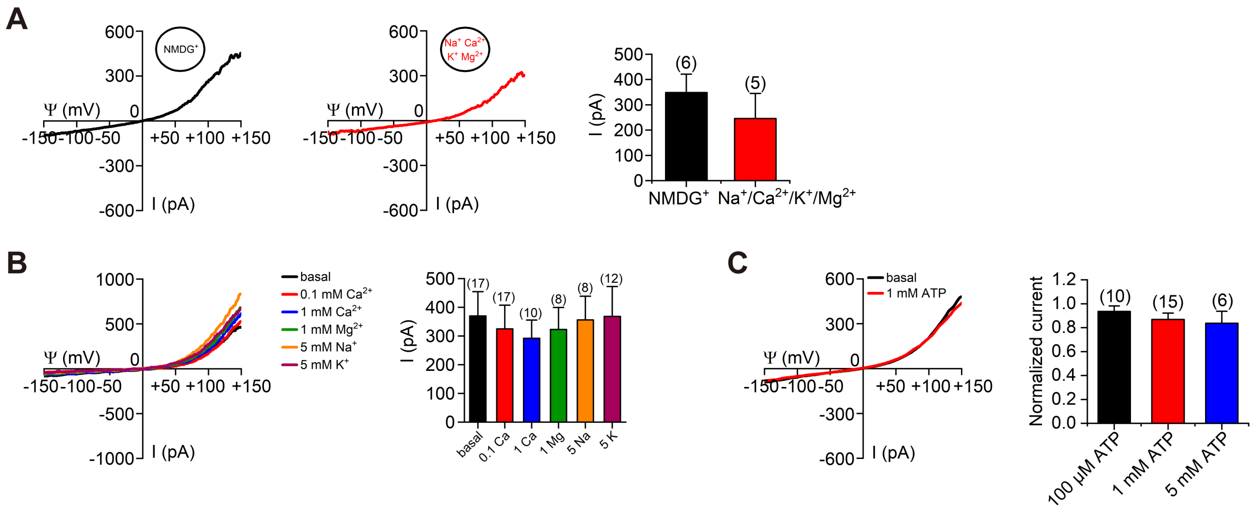

Fig. S6. Cations and ATP have no effect on CLN7-mediated chloride currents.

Whole-endolysosomal Cl^- currents were recorded from HEK293T cells transfected with EGFP-CLN7. (**A**) Whole-endolysosomal currents recorded with a pipette solution containing 154 mM $NMDG^+$ (left) or containing 1 mM Ca^{2+}, 1 mM Mg^{2+}, 75 mM Na^+, and 75 mM K^+ (middle). (right) Averaged current amplitudes (at +150 mV) in different ionic conditions as shown in left and middle panels. (**B**) Representative whole-endolysosomal currents recorded in bath solutions containing the indicated cations (left). Averaged current amplitudes (at +150 mV) recorded in the presence of different cations are in (right). (**C**) Whole-endolysosomal currents recorded with or without 1 mM of ATP in the bath solution (left). (right) Currents amplitudes (at +150 mV) recorded after application of ATP, normalized to the basal currents.

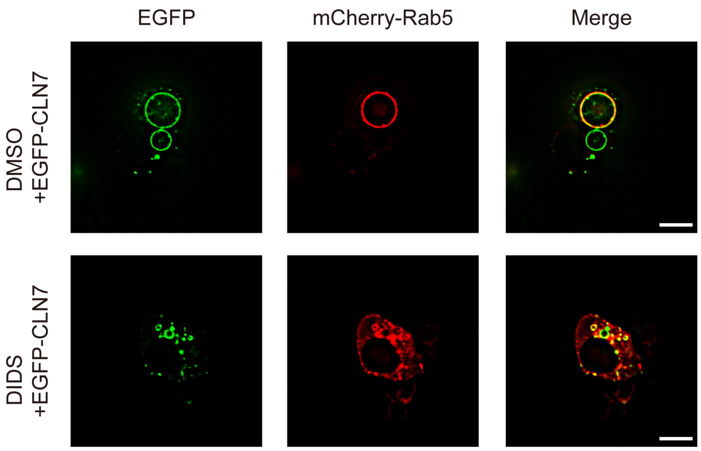

Fig. S7. Chloride channel blocker DIDS prevents CLN7-overexpression-induced enlargement of endolysosomes.

HEK293T cells were transfected with EGFP-CLN7 and mCherry-Rab5, and incubated with DMSO or 100 μM DIDS. Scale bars = 10 μm.

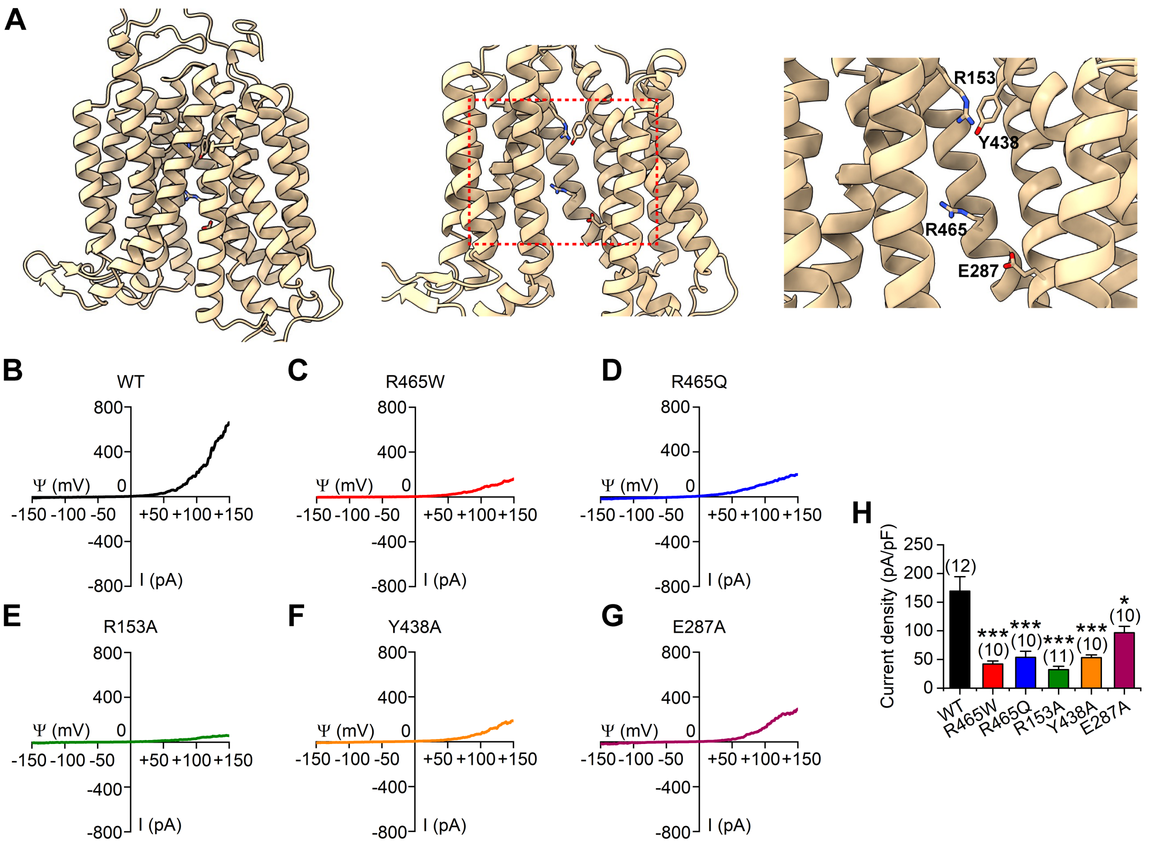

Fig. S8. Mutations in the pore region of CLN7 impair chloride conductance.

(**A**) Four potentially important amino acid sites in the predicted central pore region of CLN7 protein. The 3D structure of human CLN7 protein was predicted by AlphaFold. In order to show the amino acid sites, an α-helix is hidden in the middle and right diagrams. (**B-G**) Representative endolysosomal currents recorded in HEK293T cells transfected with N-terminally EGFP tagged wild type CLN7 (**B**), CLN7-R465W (**C**), CLN7-R465Q (**D**), CLN7-R153A (**E**), CLN7-Y438A (**F**) or CLN7-E287A (**G**). (**H**) Statistics of the current densities of (**B-G**). Data were presented as mean ± SEM.

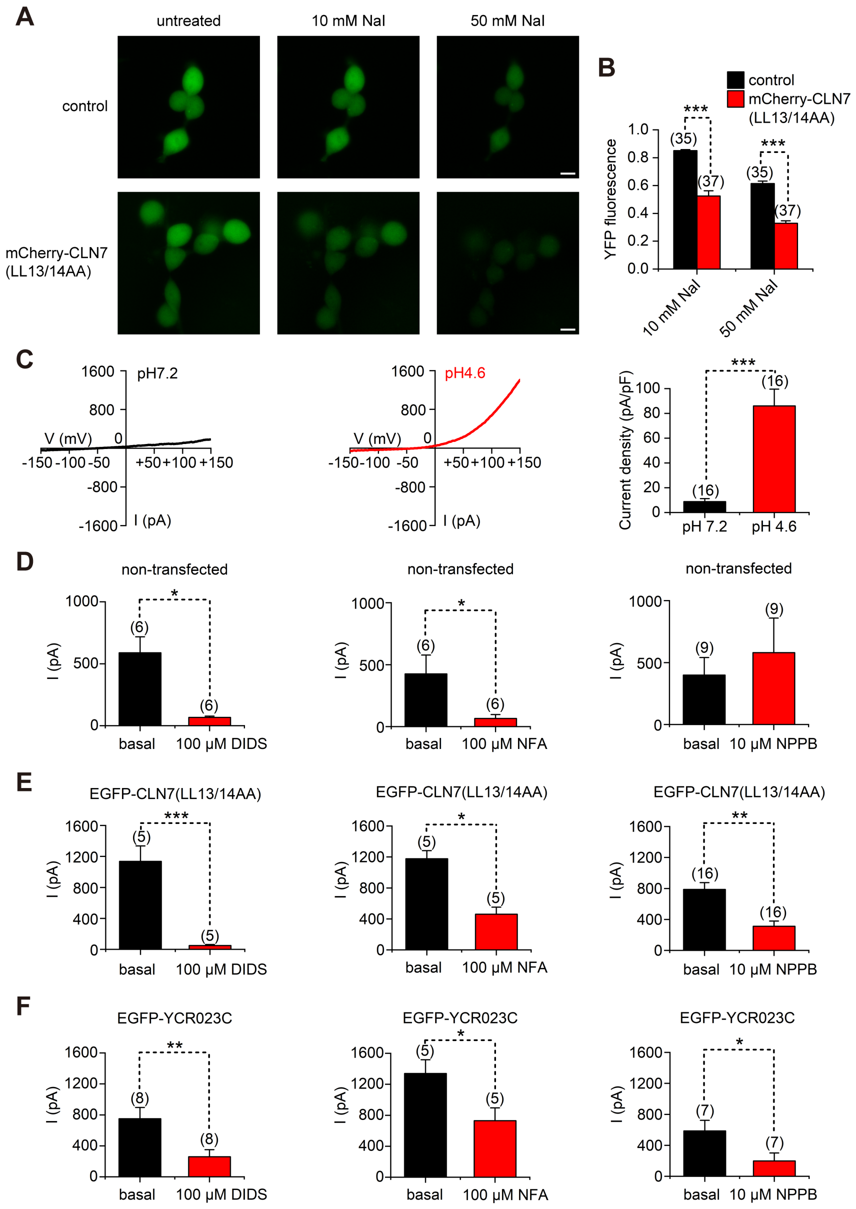

Fig. S9. Properties of the PM-targeting CLN7 mutant and yeast CLN7 homolog.
(**A**) Fluorescence of HEK293T cells co-expressing the iodide-sensitive EYFP-H148Q/I152L variant and mock vector (control; upper) or mCherry tagged CLN7-LL13/14AA (lower) before and after NaI treatment. Scale bars = 10 µm. (**B**) Fluorescent intensities of cells after treatment with 10 or 50 mM of NaI, normalized to those measured before treatment. (**C**) Whole-cell Cl^- currents recorded in EGFP-CLN7-LL13/14AA-transfected HeLa cells in bath solutions with a pH of 7.2 or 4.6. (Right) Averaged current densities measured at +150 mV. (**D**) Whole-cell Cl^- currents recorded in non-transfected HeLa cells before and after bath application of DIDS, NFA, or NPPB. (**E**) Whole-cell Cl^- currents recorded in HeLa cells expressing EGFP-CLN7-LL13/14AA after bath application of DIDS, NFA, or NPPB. (**F**) Whole-cell Cl^- currents recorded in HeLa cells expressing EGFP-YCR023C after bath application of DIDS, NFA, or NPPB.

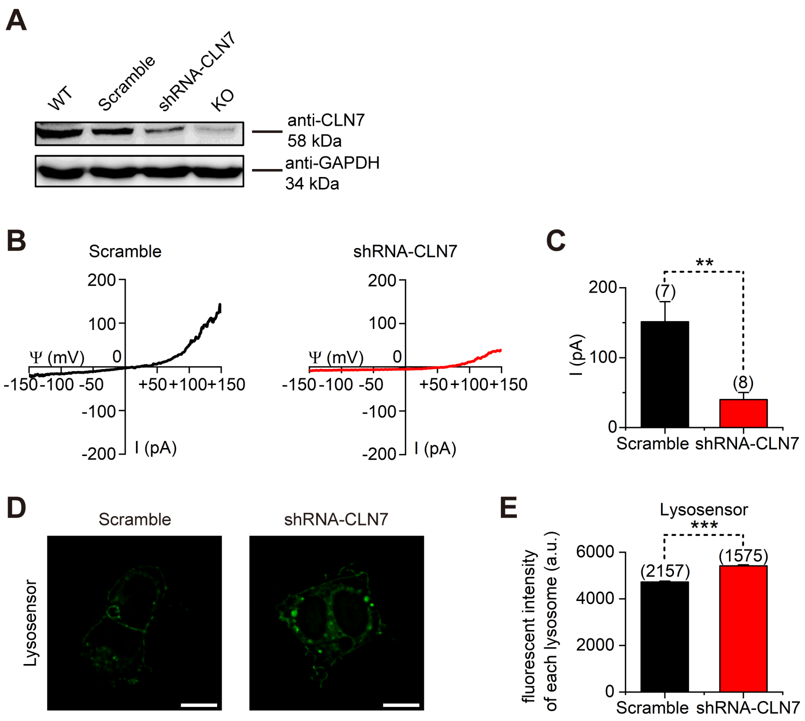

Fig. S10. Knockdown of CLN7 reduces Cl^- currents and affects lysosomal pH.

(**A**) Immunoblotting analysis shows the protein levels of CLN7 in WT, scrambled control, CLN7 knockdown and knockout HEK293T cells. (**B**) Whole-endolysosomal currents recorded from scrambled-control (left) or CLN7-knockdown (right) HEK293T cells. (**C**) Averaged current amplitudes (at +150 mV) of (**B**). (**D**) Lysosomes labeled with LysoSensor in scrambled-control (left) and CLN7-knockdown (right) HEK293T cells. Scale bars = 10 µm. (**E**) Statistical analysis of LysoSensor fluorescent intensities of lysosomes in cells shown in (**D**). For scrambled-control cells, n = 2157 lysosomes from 68 cells performed on 1 passage. For CLN7-knockdown cells, n = 1575 lysosomes from 57 cells performed on 1 passage.

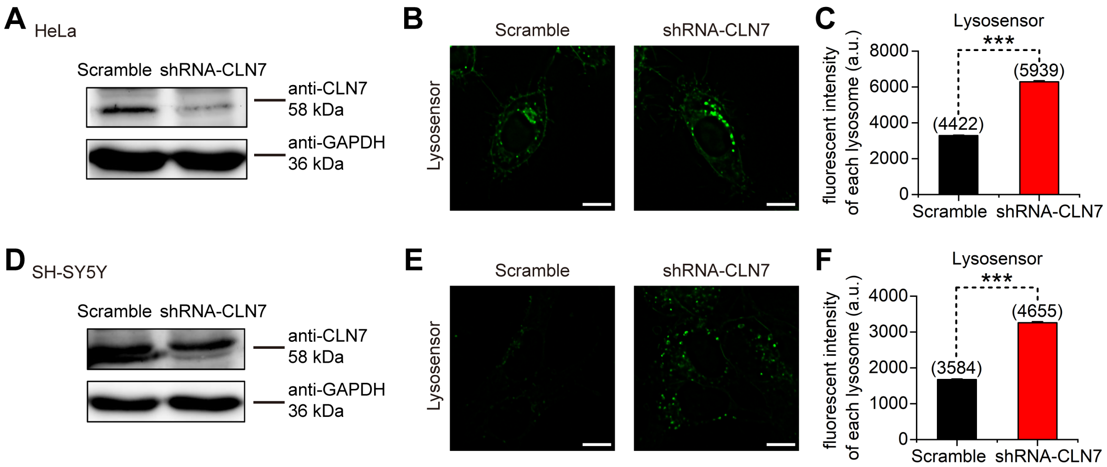

Fig. S11. Knockdown of CLN7 affects lysosomal pH in HeLa and SH-SY5Y cells.
(**A**) Immunoblotting analysis shows reduced CLN7 protein level in CLN7-knockdown HeLa cells compared to those in scrambled-control cells. (**B**) Lysosomes labeled with LysoSensor in scrambled-control (left) and CLN7-knockdown (right) HeLa cells. Scale bars = 10 µm. (**C**) Statistical analysis of LysoSensor fluorescent intensities of lysosomes shown in (**B**). For scrambled-control cells, n = 4422 lysosomes from 63 cells performed on 1 passage. For CLN7-knockdown cells, n = 5939 lysosomes from 80 cells performed on 1 passage. (**D**) Immunoblots for CLN7 expression levels in scrambled-control and CLN7-knockdown SH-SY5Y cells. (**E**) Lysosomes labeled with LysoSensor in scrambled-control (left) and CLN7-knockdown (right) SH-SY5Y cells. Scale bars = 10 µm. (**F**) Statistical analysis of LysoSensor fluorescent intensities of lysosomes shown in (**E**). For scrambled-control cells, n = 3584 lysosomes from 126 cells performed on 1 passage. For CLN7-knockdown cells, n = 4655 lysosomes from 117 cells performed on 1 passage.

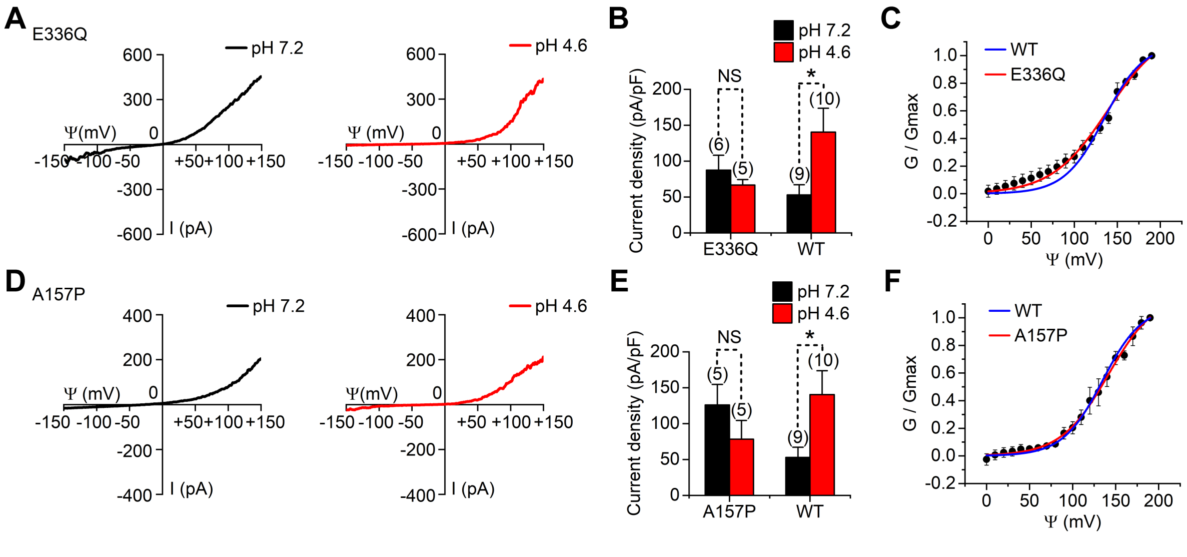

Fig. S12. Pathogenic mutations affect the pH sensitivity of CLN7 without affecting the voltage dependence.

(**A** and **D**) Representative endolysosomal currents recorded using pipette solutions with a pH of 7.2 or 4.6 in HEK293T cells expressing EGFP-CLN7-E336Q (**A**) or EGFP-CLN7-A157P (**D**). The currents were induced using a ramp protocol (−150 to +150 mV in 1 s, Vh = 0 mV). (**B** and **E**) Current densities measured at +150 mV under conditions used in (**A** and **D**). The WT data are replotted from Fig. 3H for comparison. (**C** and **F**) The voltage-dependent activation curves for CLN7-E336Q (**C**) and CLN7-A157P (**F**). The G values were calculated from the current amplitudes recorded using 500-ms depolarizing steps (0 to +190 mV, 10 mV step; Vh = −70 mV). The WT data are replotted from Fig. 2J for comparison. Data are presented as the mean ± SEM.

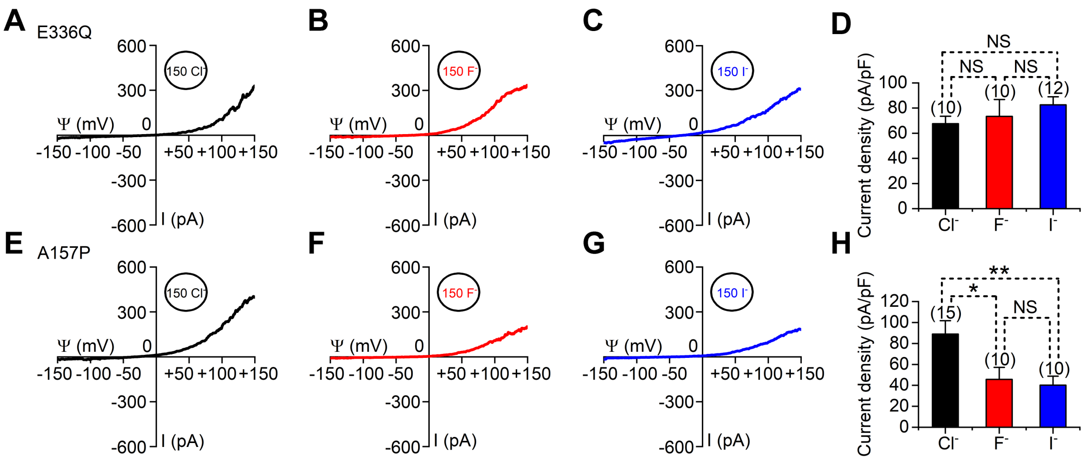

Fig. S13. Pathogenic mutations of CLN7 affect its selectivity to halide ions.

(A-H) Whole-endolysosomal currents were recorded using a ramp protocol (−150 to +150 mV in 1 s, Vh = 0 mV) from HEK293T cells expressing EGFP-CLN7-E336Q (**A-D**) or EGFP-CLN7-A157P (**E-H**). (**A-C**) Representative CLN7-E336Q currents recorded with pipette solutions containing 150 mM Cl^- (**A**), 150 mM F^- (**B**), or 150 mM I^- (**C**). (**D**) Current densities measured at +150 mV under conditions used in (**A-C**). (**E-G**) Representative CLN7-A157P currents recorded with pipette solutions containing 150 mM Cl^- (**E**), 150 mM F^- (**F**), or 150 mM I^- (**G**). (**H**) Current densities measured at +150 mV under conditions used in (**E-G**). Data are presented as the mean ± SEM.

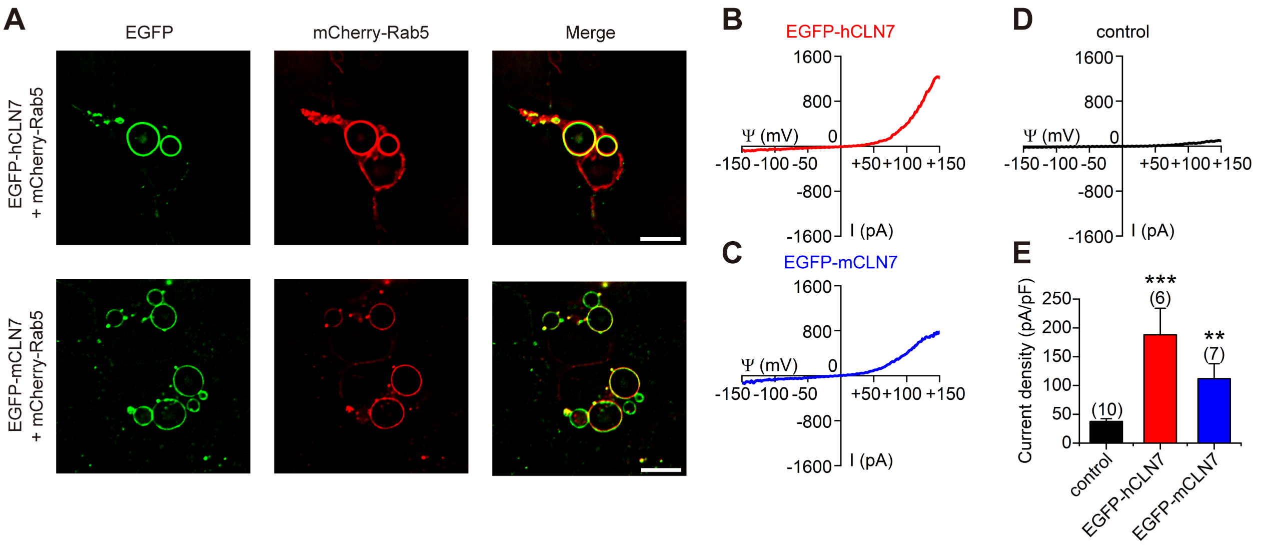

Fig. S14. Human CLN7 and mouse CLN7 exhibit similar properties in endolysosomes. (**A**) Colocalization of EGFP-tagged human CLN7 (EGFP-hCLN7) or mouse CLN7 (EGFP-mCLN7) with mCherry-tagged Rab5 in HEK293T cells. Scale bars = 10 µm. (**B-E**) Representative whole-endolysosomal Cl^- currents recorded from HEK293T cells transfected with human (**B**) or mouse CLN7 (**C**), as well as from non-transfected cells (control) (**D**). (**E**) Averaged peak current densities (measured at +150 mV) from cells with different transfections. Data are presented as the mean ± SEM.

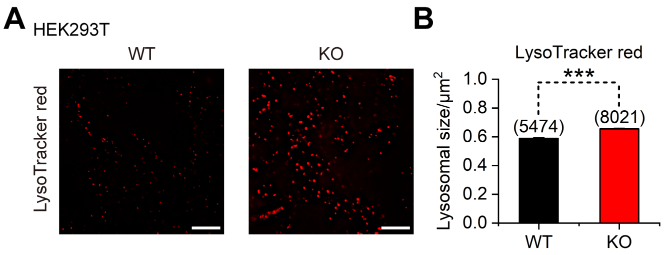

Fig. S15. Knockout of CLN7 affects lysosomal size in HEK293T.

(**A**) Representative images of WT and CLN7-knockout (KO) HEK293T cells stained with LysoTracker red. Scale bars = 10 µm. (**B**) Lysosome sizes shown as the area of the lysosomes in cells represented in (**A**). For WT cells, n = 5474 lysosomes from 188 cells performed on 1 passage. For KO cells, n = 8021 lysosomes from 210 cells performed on 1 passage.

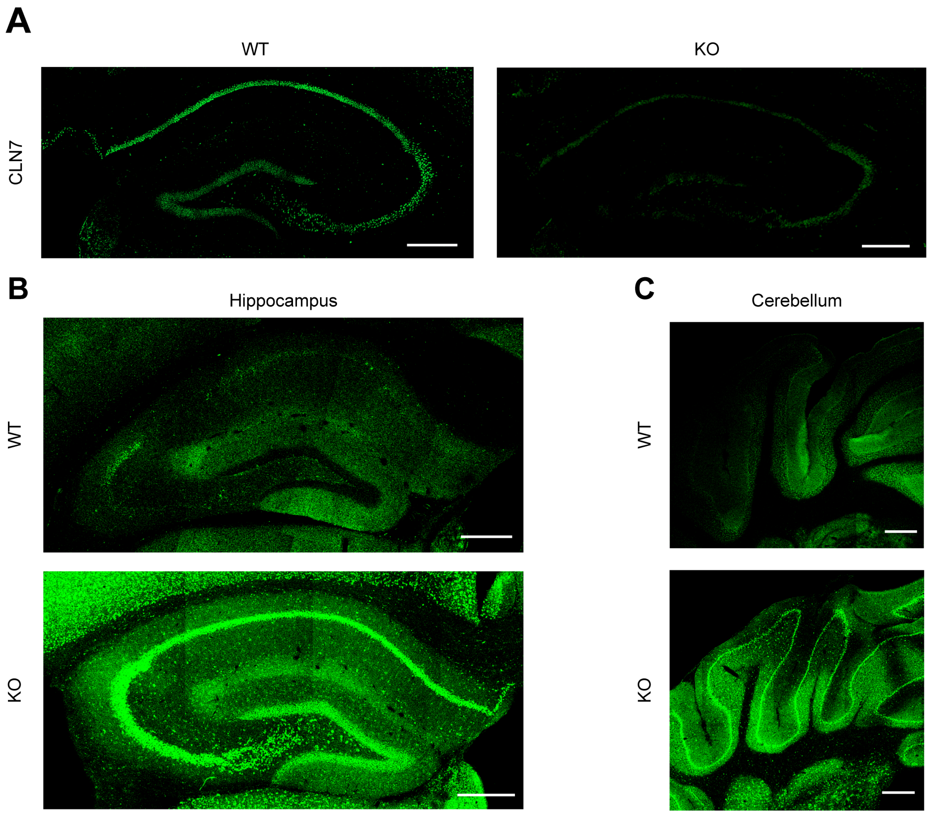

Fig. S16. Immunostaining of CLN7 and autofluorescence in brain tissues from WT and Cln7-KO mice.

(**A**) Immunofluorescence assay of CLN7 in hippocampal slices from nine-month-old WT and CLN7-KO mice. Scale bars = 200 μm. (**B**) Autofluorescence of hippocampal slices from nine-month-old WT and CLN7-KO mice. Scale bars = 200 μm. (**C**) Autofluorescence of cerebellar slices from nine-month-old WT and CLN7-KO mice. Scale bars = 200 μm.

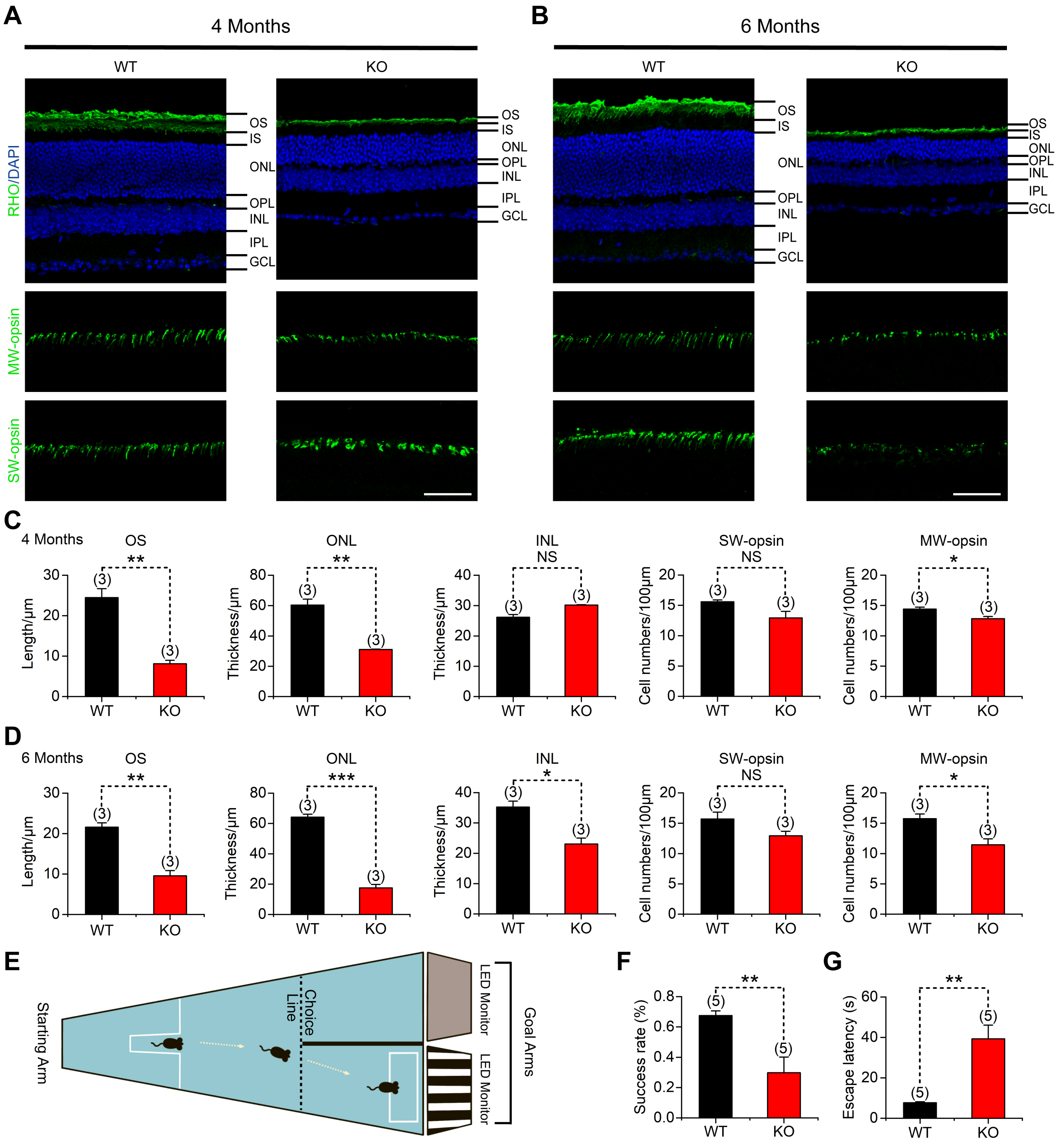

Fig. S17. Degeneration of photoreceptor cells and visual impairment in Cln7-KO mice. (**A** and **B**) Retinas form four-month-old (**A**) and six-month-old (**B**) WT and CLN7-KO mice were stained with antibodies against marker proteins for rods (rhodopsin, RHO) or cones (MW-opsin and SW-opsin). DAPI staining was used to observe the nuclei of the cells. Scale bar = 50 µm. Abbreviations: OS, outer segment; IS, inner segment; ONL, outer nuclear layer; OPL, outer plexiform layer; INL, inner nuclear layer; IPL, inner plexiform layer; GCL, ganglion cell layer. (**C** and **D**) Quantification of rods and cones outer segment length, ONL thickness and INL thickness from four-month-old (**C**) and six-month-old (**D**) mice. (**E**) Schematic diagram of the visual water maze. (**F**) Success rate of WT and CLN7-KO mice in the visual water maze. (**G**) Mean escape latency to the platform of WT and CLN7 KO mice. Data are presented as the mean ± SEM.

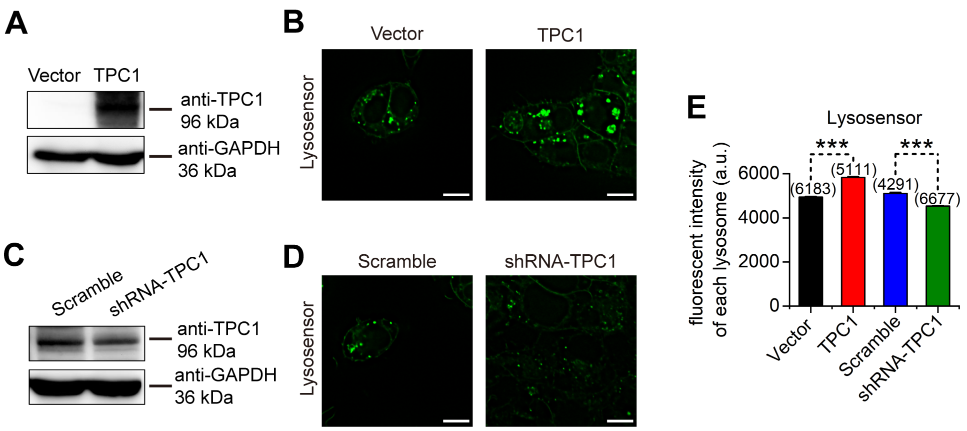

Fig. S18. Overexpression and knockdown of TPC1 affect lysosomal pH.

(**A**) Immunoblotting analysis of TPC1 in HEK293T cells transfected with mock vector or human TPC1. (**B**) Lysosomes labeled with LysoSensor in HEK293T cells transfected with mock vector or human TPC1. Scale bars = 10 µm. (**C**) Immunoblotting analysis of TPC1 in scrambled control and TPC1-knockdown HEK293T cells. (**D**) Lysosomes labeled with LysoSensor in scrambled-control and TPC1-knockdown HEK293T cells. Scale bars = 10 µm. (**E**) Statistical analysis of LysoSensor fluorescent intensities of lysosomes shown in (**B** and **D**). The lysosomes were from 190 cells expressing mock vector, 203 cells expressing TPC1, 183 cells expressing scrambled shRNA, and 196 cells expressing shRNA against TPC1. The cells in each group were from same passage. Data are presented as the mean ± SEM.

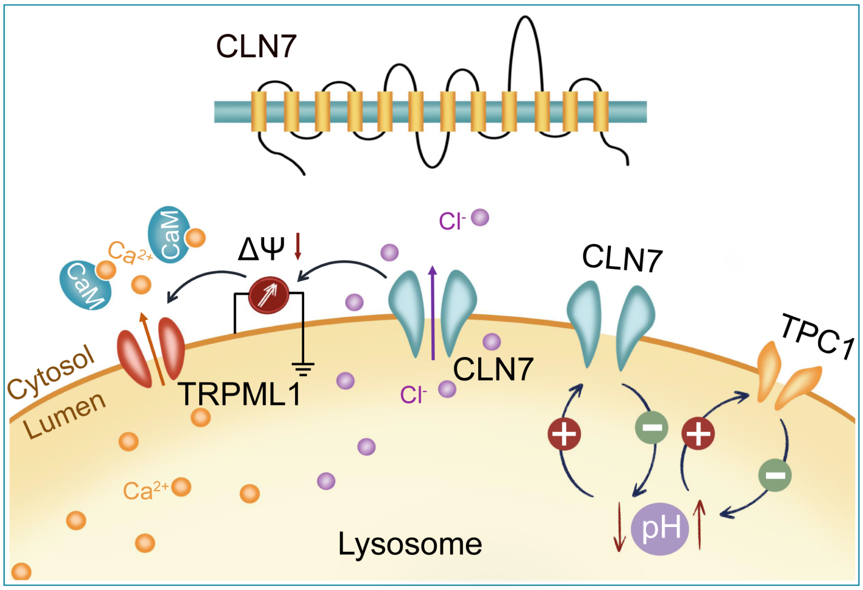

Fig. S19. An overview model illustrating the roles of CLN7 in lysosomal physiology.

The twelve-transmembrane protein CLN7 forms a chloride channel on lysosomal membrane and mediates the movement of chloride ions from the lysosomal lumen to the cytosol. CLN7 regulates lysosomal membrane potential, promotes the release of lysosomal Ca^{2+} through TRPML1 and in turn activates calmodulin. In addition, CLN7 regulates lysosomal pH in a negative feedback manner, and works with another lysosomal channel TPC1 to restrict lysosomal pH to a specific range.
